# ﻿Revision of *Lathonurarectirostris* (O.F. Müller, 1785) (Anomopoda, Macrothricidae) leads to translocation of East Asian populations to a separate species, *Lathonurabekkerae* sp. nov.

**DOI:** 10.3897/zookeys.1249.154922

**Published:** 2025-08-19

**Authors:** Ivan A. Dadykin, Dmitry D. Pereboev

**Affiliations:** 1 A.N. Severtsov Institute of Ecology and Evolution of the Russian Academy of Sciences, Leninsky Prospect 33, Moscow 119071, Russia A.N. Severtsov Institute of Ecology and Evolution of the Russian Academy of Sciences Moscow Russia

**Keywords:** Barcoding, Cladocera, male, morphology, new species, taxonomy, zooplankton

## Abstract

The family Macrothricidae (Branchiopoda: Anomopoda) remains one of the least studied groups of water fleas (Cladocera). One of macrothricids with an unclear phylogenetic status is *Lathonura* Lilljeborg, 1853 comprising a single universally accepted valid species, *L.rectirostris* (O.F. Müller, 1785). Despite its wide distribution in the Northern Hemisphere, no studies were conducted to prove conspecificity of *L.rectirostris* from different regions and properly describe its gamogenetic stages. Here, the morphological and genetic diversity of *Lathonura* in the Holarctic is revised. Our results show that in East Eurasia and Alaska a separate species of the genus occurs, *L.bekkerae***sp. nov.**, which differs from *L.rectirostris* s. str. by the posteroventral valve armature, structure of antenna I, and ephippium ornamentation. Mitochondrial DNA barcoding supports separation of *L.bekkerae***sp. nov.** and reveals a deeply divergent clade of *L.rectirostris* s. l. in Canada, for which parthenogenetic females are morphologically indistinct from those of *L.rectirostris* s. str. *Lathonura* distribution fits well to the known patterns of Anomopoda biogeography. Males of *Lathonura* possess two additional setae at antenna II basipodite, P1 with a subdistal lobe lacking setae, P1 IDL with a hook and an additional seta, and an unmodified postabdomen. As noted in some previous studies, *Lathonura* likely represents a phylogenetic lineage distinct from the subfamily Macrothricinae, differing from most macrothricines by the absence of Fryer’s fork at P1 inner endite anterior setae and presence of P1 accessory seta. However, the phylogenetic position of the genus and its diversity in South Eurasia, Africa, and North America requires further studies.

## ﻿Introduction

Anomopoda Sars, 1865 (Crustacea: Cladocera) is a widespread group of aquatic crustaceans playing a pivotal role in continental waters (Błedzki and Rybak 2016; Korovchinsky et al. 2021). Several anomopod species are used in aquaculture, bioassays, and are accepted widely as model organisms for evolutionary biology and ecology (Shaw et al. 2008; Smirnov 2017; Ebert 2022; Qiu et al. 2022). However, this group remains insufficiently studied in many aspects, including taxonomy of several groups. Despite great efforts performed in this field in recent years (Forró et al. 2008; Macêdo et al. 2024), particularly in Daphniidae (Popova et al. 2016; Bekker et al. 2018; Zuykova et al. 2019; Garibian et al. 2020; Kotov et al. 2021; Pereboev et al. 2025), Moinidae (Bekker et al. 2016; Alonso et al. 2019; Makino et al. 2020), Eurycercidae (Bekker et al. 2012; Bekker and Kotov 2016), and Chydoridae (Van Damme and Dumont 2008; Belyaeva and Taylor 2009; Van Damme et al. 2011; Sinev et al. 2017; Sousa and Elmoor-Loureiro 2018), several anomopod families still lack taxonomic revisions, which is particularly true for the family Macrothricidae Norman & Brady, 1867 (Dumont and Silva-Briano 1998).

This relatively small family of water fleas comprises ~80 recent species belonging to 11 genera, distributed worldwide (Smirnov 1992; Kotov et al. 2013; Rogers et al. 2019). Due to their morphological diversity and complexity, macrothricids remained out of scope of most researchers for a long time (Dumont and Silva-Briano 1998; Kotov et al. 2004; Neretina and Kotov 2017a). Moreover, most Macrothricidae are associated with particular types of water bodies and substrates (Fryer 1974; Smirnov 1992), which makes them hard to find with classical sampling methods. Within the family, various adaptations for living on/in substrate are observed, e.g., development of a strong burrowing spine on the antenna II, specific armature of ventral valve margin, transformation of postabdomen shape. (Fryer 1974; Kotov 2006). One of the most striking examples of such specialization is *Lathonura* Lilljeborg, 1853, a conspicuous genus adapted to crawling and attachment to substrates due to specific structure of its ventral valve margins and thoracic limbs (Sergeev 1971; Fryer 1974; Dumont and Silva-Briano 1998).

**Figure 1.**
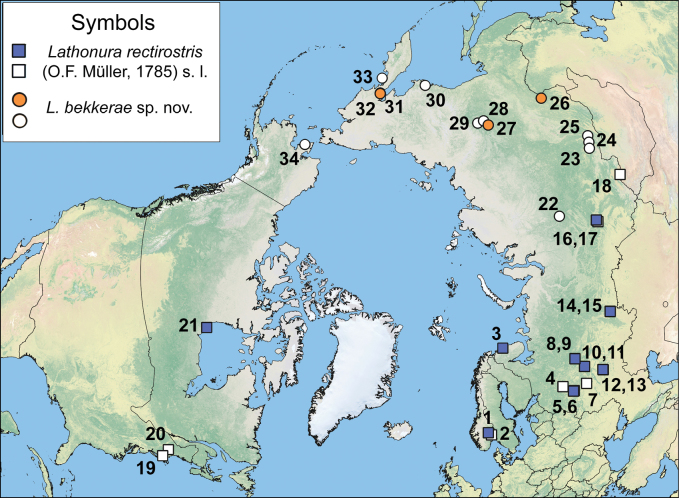
Studied populations of *Lathonura* Lilljeborg, 1853. Squares indicate records of *L.rectirostris* (O.F. Müller, 1785) s. l., circles are records of *L.bekkerae* sp. nov. Filled points are included in the genetic analysis (see Table 1); for white points, only morphological analysis was conducted. Localities: 1, 2, Innlandet, Norway (60°47.70'N, 10°8.02'E; 60°31.77'N, 10°17.67'E); 3, Murmansk Area, Russia (69°11.80'N, 35°7.31'E); 4, Tver Area, Russia (57°36.55'N, 35°54.84'E); 5, Moscow Area, Russia (55°45.11'N, 36°30.65'E); 6, Moscow Area, Russia (55°41.93'N, 36°43.83'E); 7, Ryazan Area, Russia (54°34.95'N, 40°3.19'E); 8, 9, Kostroma Area, Russia (58°10.15'N, 44°30.57'E; 58°11.3'N, 44°33.24'E); 10, 11, Nizhny Novgorod Area, Russia (56°12.69'N, 43°45.13'E; 56°12.32'N, 43°45.41'E); 12, 13, Penza Area, Russia (53°14.04'N, 45°5.28'E; 53°10.36'N, 45°4.46'E); 14, 15, Chelyabinsk Area, Russia (55°0.68'N, 59°54.48'E; 54°59.46'N, 59°50.38'E); 16, 17, Tomsk Area, Russia (56°43.33'N, 84°41.52'E; 56°32.63'N, 84°9.48'E); 18, Uvs Aimag, Mongolia (50°29.67'N, 93°36.02'E); 19, Rhode Island, U.S.A. (41°55.45'N, 72°13.04'W); 20, New Hampshire, U.S.A. (43°22.54'N, 73°55.80'W); 21, Manitoba, Canada (58°42.54'N, 94°9.66'W); 22, Krasnoyarsky Krai, Russia (62°21.24'N, 89°2.49'E); 23–25, Irkutsk Area, Russia (52°58.28'N, 102°38.81'E; 52°29.06'N, 103°57.22'E; 52°1.64'N, 105°24.76'E); 26, Zabaikalsky Krai, Russia (53°29.20'N, 120°3.04'E); 27–29, Yakutia, Russia (62°12.28'N, 127°44.84'E; 62°1.11'N, 129°45.42'E; 62°48.36'N, 131°4.50'E); 30, Magadan Area, Russia (59°44.37'N, 150°51.36'E); 31–33, Kamchatsky Krai, Russia (61°17.58'N, 164°42.30'E; 61°22.62'N, 164°44.28'E; 58°52.58'N, 163°48.55'E); 34, Alaska, U.S.A. (65°5.34'N, 165°4.63'W). For more information on localities, see Suppl. material 6.

**Figure 2.**
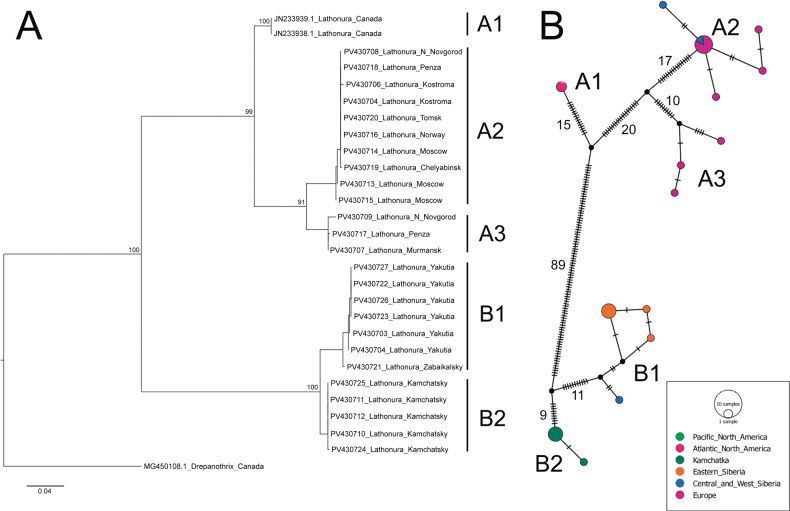
Genetic diversity of *Lathonura* Lilljeborg, 1853 revealed by COI gene Folmer fragment barcoding. A. Maximum likelihood phylogram (branch supports obtained by 100 standard bootstrap replicates); B. TCS haplotype network.

**Figure 3.**
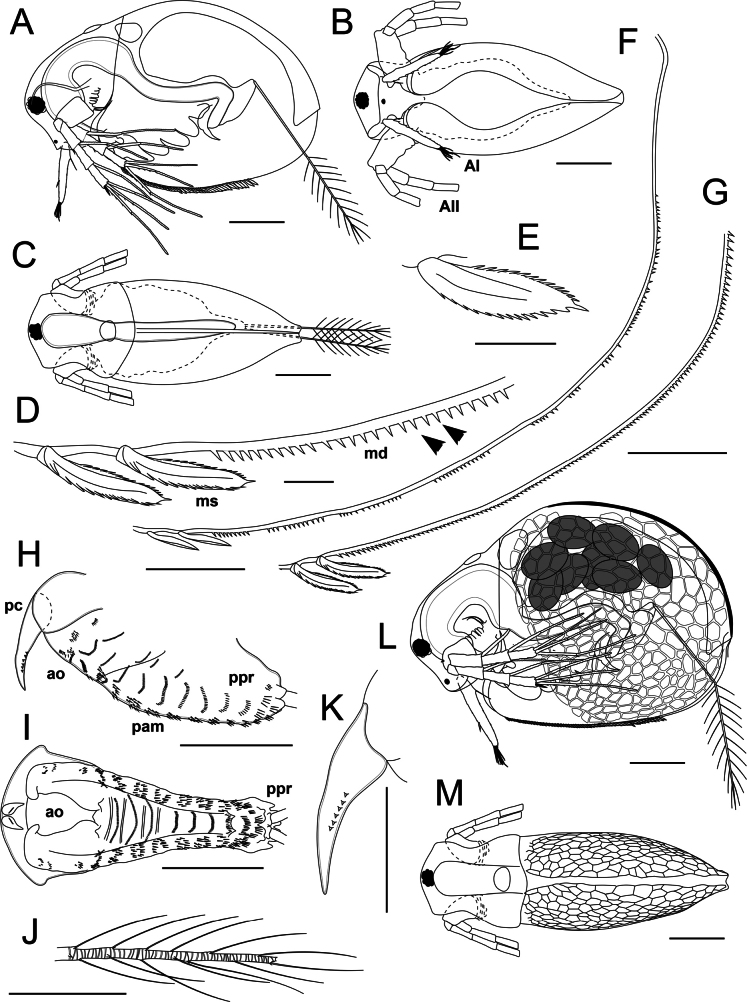
*Lathonurarectirostris* (O.F. Müller, 1785) from Lake Randsfjorden, Innlandet, Norway (Fig. 1, loc. 1), gross morphology. A–K. Parthenogenetic female: A. General lateral view; B. Ventral view; C. Dorsal view; D–G. Posteroventral valve armature; H. Postabdomen in lateral view; I. Postabdomen in dorsal view; J. Postabdominal seta tip; K. Postabdominal claw; L, M. Ephippial female: L. General lateral view; M. Dorsal view. Abbreviations: AI, antenna I; AII, antenna II; ao, anal opening; mas, marginal setae of valve; md, marginal denticles; pam, preanal margin of postabdomen; pc, postabdominal claw; ppr, postabdominal process. Scale bars: 200 µm (A–C, L, M); 10 µm (D, E); 50 µm (F, G, J, K); 100 µm (H, I).

Despite that, to date, *Lathonura* includes only a single universally accepted valid species (Kotov et al. 2013), synonymy for this genus is rather complicated. Representatives of the genus were described initially as *Daphniarectirostris* O.F. Müller (1785: pl. XII, figs 1–3). Later, Koch (1841) emended the genus *Pasithea* Koch, 1841 which was subsequently replaced by *Lathonura* (Lilljeborg 1853), as the name *Pasithea* had been used already for a member of Arachnida Lamarck, 1801 (see Leydig 1860). At least two taxa of *Lathonura* (*L.spinosa* Schödler, 1858 and *L.lacustris* Leydig, 1860, both described from Germany) were first considered as independent species and were subsequently synonymized with *L.rectirostris* (O.F. Müller, 1785) (Smirnov 1992; Korovchinsky et al. 2021). Also, Cosmovici (1901) established L.rectirostrisvar.dorsispina Cosmovici, 1901 based on his material from Beldiman, Romania, noting that this could be a new taxon. However, the author did not provide illustrations and formal description for this material, which thus must be placed in nomina nuda. One more *Lathonura* species, *L.ovalis* Mahoon & Sabjr, 1985, was described from Changa Manga in Punjab, Pakistan based on the shape of the antenna I and postabdominal claw, eye shape, and number of anal spines, but the description was very brief; also, available figures are not consistent with some of the abovementioned diagnostic features (Mahoon and Sabjr 1985). In this respect, both *L.dorsispina* and *L.ovalis* are now considered as species inquirendae (Kotov et al. 2013), in need of clarification with new material.

In contrast, representatives of the type (and the only undoubtedly valid) species, *Lathonurarectirostris*, were redescribed and illustrated in numerous studies (e.g., Herrick 1884; Lilljeborg 1900; Sramek-Hušek et al. 1962; Manuilova 1964; Flössner 1972; Hudec 2010; Rogers et al. 2019; inter alia). These descriptions were focused mostly on a gross morphology. Thoracic limbs of *L.rectirostris* were thoroughly described by Fryer (1974) and, to a lesser extent, by Sergeev (1971) and Silva-Briano (1998). Also, Sergeev (1971, 1973) and Fryer (1974) provided some data on the functional morphology of *L.rectirostris*. Formation of resting eggs and ephippium were studied by Makrushin (1970) and Fryer (1972). However, gamogenetic stages of *Lathonura* received relatively little attention, despite the fact that these stages are known to be important for identification in several anomopod groups, including some Macrothricidae (Kotov 2008, 2013). Brief notes on male morphology were provided by Gruber and Weismann (1877), Birge (1879), Herrick (1884), Lilljeborg (1900), Sramek-Hušek et al. (1962), Manuilova (1964), and Flössner (1972), but the available illustrations are mostly not informative for a final conclusion on a real species number within the genus.

Nowadays, *Lathonurarectirostris* is considered as a widely distributed species, being recorded throughout the temperate zone of Eurasia and North America (Rogers et al. 2019) and even in South Africa (Hart and Dumont 2005). However, no taxonomic or phylogeographic studies were conducted to prove conspecificity of *L.rectirostris* in different parts of its spacious range. It should be noted that many anomopod taxa previously had been supposed to have a wide distribution were subsequently revised and proved to be groups of closely related taxa or deeply divergent genetic lineages (Belyaeva and Taylor 2009; Xu et al. 2009; Millette et al. 2011; Bekker et al. 2016, 2018; Garibian et al. 2020, 2021; Makino et al. 2020; Kotov et al. 2021; Zuykova et al. 2022).

Recently Dadykin et al. (2025) reported that L.cf.rectirostris from Karaginsky Island, northeast Kamchatka Peninsula (Russia), show some morphological differences from European populations. However, the aforementioned study contained only a brief description, and the morphological characters were not compared with those of other East Asian and American populations of *Lathonura*, and the status of L.cf.rectirostris from Kamchatka remains unclear. Apparently, morphology, diversity, and distribution of the genus *Lathonura* require further studies. Here, we aim to summarize recent knowledge on *L.rectirostris* in the Holarctic and conduct a revision for the species based on morphological and genetic data from Palearctic populations mainly.

## ﻿Materials and methods

### ﻿Sample collection

Samples were collected using a small throw net (25 cm diameter, 30 µm mesh size) from a number of localities in Europe, Siberia, Mongolia, the Russian Far East, and Alaska in 2004–2023 (Fig. 1, Suppl. material 6). Most of the studied samples were preserved in 96% ethanol and then stored at 4 °C. For morphological analysis, we used several samples preserved in 3% formaldehyde. The list of investigated specimens is provided in Suppl. material 6 (see also Fig. 1). Type specimens are deposited at the collection of the Zoological Museum of Lomonosov Moscow State University (**MSU**), Moscow, Russia (further abbreviated as MGU MI-##), and at the working collection of the laboratory of aquatic ecology and invasions, A.N. Severtsov Institute of Ecology and Evolution of the Russian Academy of Sciences (**IEE**), Moscow, Russia (AAK-##), as all original samples.

### ﻿Genetic analysis

Prior to genetic analysis, specimens were identified to species level using a binocular optical microscope to avoid mistakes. DNA of single specimens was extracted using a Wizard Genomic DNA Purification Kit (Promega Corporation, Madison, WI, U.S.A.). Each individual was dried at room temperature, then placed in the lysis medium (100 µl of Nuclei Lysis Solution, 20 µl of EDTA, and 10 µl of protein kinase K), crushed by sterile plastic pestle and incubated at 55 °C for 18–20 hours. At the next step, 125 µl of SV Lysis Buffer were added to each specimen, the samples were mixed in a vortex and immediately transferred to minicolumns. After 10 min of incubation at room temperature, the samples were centrifuged for 3 min at 13000 rpm. Then 650 µl of diluent was added to each sample, with two rounds of centrifugation (3 min at 13000 rpm and 2 min at 13000 rpm). The dried minicolumns were transferred to a new microtube. Elution was repeated twice: on each step, 30 µl of distilled water (T = 55 °C) were added to a minicolumn, incubated for 10 min at room temperature and centrifuged for 3 min at 13000 rpm. The extracted DNA was stored at -20 °C.

For barcoding, mitochondrial COI gene Folmer fragment (~700 bp length) was amplified, using two pairs of primers: dg_ctd_folmer_F (5’-TCRACYAATCATAAGGAYATTGG-3’) as a forward primer and simoR (5’-CCTCTYTCATGACTAATAATATG-3’) as a reverse primer (original), or dg_ctd_folmer_F / HCO2198 (5’-CCTCTYTCATGACTAATAATATG-3’, Folmer et al. 1994) pair, respectively (Table 1). For PCR, the Encyclo PCR kit was applied (Eurogene, Russia). PCR was performed in total volume of 25 µl (5 μl of HS-Encyclo Buffer, 14 μl of distilled water, 2 μl of forward primer, 2 μl of reverse primer, and 2 μl of DNA template). Conditions of PCR were set as follows: 95 °C for 5 min; 5 cycles: 95 °C for 30 sec, 48 °C for 50 sec, 72 °C for 1 min; 34 cycles: 95 °C for 30 sec, 50 °C for 50 sec, 72 °C for 1 min; 72 °C for 5 min. Size of the PCR products was verified by 1% agarose gel electrophoresis. The PCR products were purified by CleanMag DNA PCR kit (Eurogene, Russia). Two-directional Sanger sequencing was performed by Syntol Ltd. (Russia).

**Table 1.** List of *Lathonura* sequences included in the genetic analysis. Locality numbers correspond to Fig. 1 and Suppl. material 6.

Species	GenBank accession number	Locality number	Region and country	Primer pair	Sex	Source
*Lathonurarectirostris* (O.F. Müller, 1785) s. str.	PV430716	2	Innlandet, Norway	dg_ctd_folmer_F / HCO2198	ephippial female	original
*L.rectirostris* (O.F. Müller, 1785) s. str.	PV430707	3	Murmansk Area, Russia	dg_ctd_folmer_F / HCO2198	parthenogenetic female	original
*L.rectirostris* (O.F. Müller, 1785) s. str.	PV430713	5	Moscow Area, Russia	dg_ctd_folmer_F / simoR	parthenogenetic female	original
*L.rectirostris* (O.F. Müller, 1785) s. str.	PV430714	5	Moscow Area, Russia	dg_ctd_folmer_F / simoR	parthenogenetic female	original
*L.rectirostris* (O.F. Müller, 1785) s. str.	PV430715	5	Moscow Area, Russia	dg_ctd_folmer_F / HCO2198	parthenogenetic female	original
*L.rectirostris* (O.F. Müller, 1785) s. str.	PV430705	8	Kostroma Area, Russia	dg_ctd_folmer_F / HCO2198	parthenogenetic female	original
*L.rectirostris* (O.F. Müller, 1785) s. str.	PV430706	9	Kostroma Area, Russia	dg_ctd_folmer_F / HCO2198	parthenogenetic female	original
*L.rectirostris* (O.F. Müller, 1785) s. str.	PV430708	11	Nizhny Novgorod Area, Russia	dg_ctd_folmer_F / simoR	parthenogenetic female	original
*L.rectirostris* (O.F. Müller, 1785) s. str.	PV430709	11	Nizhny Novgorod Area, Russia	dg_ctd_folmer_F / simoR	parthenogenetic female	original
*L.rectirostris* (O.F. Müller, 1785) s. str.	PV430718	12	Penza Area, Russia	dg_ctd_folmer_F / simoR	parthenogenetic female	original
*L.rectirostris* (O.F. Müller, 1785) s. str.	PV430717	12	Penza Area, Russia	dg_ctd_folmer_F / simoR	parthenogenetic female	original
*L.rectirostris* (O.F. Müller, 1785) s. str.	PV430719	15	Chelyabinsk Area, Russia	dg_ctd_folmer_F / simoR	parthenogenetic female	original
*L.rectirostris* (O.F. Müller, 1785) s. str.	PV430720	16	Tomsk Area, Russia	dg_ctd_folmer_F / simoR	parthenogenetic female	original
*L.rectirostris* (O.F. Müller, 1785) s. l.	JN233938.1	21	Manitoba, Canada	-	parthenogenetic female	Jeffery et al. (2011)
*L.rectirostris* (O.F. Müller, 1785) s. l.	JN233939.1	21	Manitoba, Canada	-	parthenogenetic female	Jeffery et al. (2011)
*L.bekkerae* sp. nov.	PV430721	26	Zabaikalsky Krai, Russia	dg_ctd_folmer_F / simoR	parthenogenetic female	original
*L.bekkerae* sp. nov.	PV430703	27	Yakutia, Russia	dg_ctd_folmer_F / HCO2198	parthenogenetic female	original
*L.bekkerae* sp. nov.	PV430704	27	Yakutia, Russia	dg_ctd_folmer_F / HCO2198	parthenogenetic female	original
*L.bekkerae* sp. nov.	PV430722	27	Yakutia, Russia	dg_ctd_folmer_F / simoR	parthenogenetic female	original
*L.bekkerae* sp. nov.	PV430723	27	Yakutia, Russia	dg_ctd_folmer_F / simoR	parthenogenetic female	original
*L.bekkerae* sp. nov.	PV430726	27	Yakutia, Russia	dg_ctd_folmer_F / simoR	parthenogenetic female	original
*L.bekkerae* sp. nov.	PV430727	27	Yakutia, Russia	dg_ctd_folmer_F / simoR	parthenogenetic female	original
*L.bekkerae* sp. nov.	PV430725	31	Kamchatsky Krai, Russia	dg_ctd_folmer_F / simoR	parthenogenetic female	original
*L.bekkerae* sp. nov.	PV430724	31	Kamchatsky Krai, Russia	dg_ctd_folmer_F / simoR	parthenogenetic female	original
*L.bekkerae* sp. nov.	PV430710	32	Kamchatsky Krai, Russia	dg_ctd_folmer_F / simoR	parthenogenetic female	original
*L.bekkerae* sp. nov.	PV430711	32	Kamchatsky Krai, Russia	dg_ctd_folmer_F / simoR	parthenogenetic female	original
*L.bekkerae* sp. nov.	PV430712	32	Kamchatsky Krai, Russia	dg_ctd_folmer_F / simoR	parthenogenetic female	original

The resulting sequences were processed and merged into contigs using the CodonCode Aligner v. 11.0.2 (CodonCode Corporation, USA). Authenticity of the sequences was verified by BLASTn comparisons (Boratyn et al. 2013). Additional sequences were obtained from the NCBI GenBank database (Sayers et al. 2019). Then, the contigs were aligned in MEGA X (Kumar et al. 2018) using the ClustalW tool. For final alignment, a 639-bp length fragment was used. A sequence of *Drepanothrixdentata* (Eurén, 1861) from GenBank (MG450108.1) was chosen as the outgroup. A list of sequences included in the analysis is provided in Table 1 (see also Fig. 1).

The mean uncorrected p-distances (within and between species) were calculated based on the trimmed COI data using MEGA 11 for Linux (Tamura et al. 2021). Construction of a maximum likelihood phylogenetic tree and automatic estimation of the best evolutionary model were performed using the IQTREE Web Server (Trifinopoulos et al. 2016). Branch support of the clades was estimated by nonparametric bootstrap with 100 permutations calculated using IQTREE Web Server. The phylogram was visualized in the FigTree v. 1.4.4 software. TCS haplotype network (Clement et al. 2002) was constructed and visualized in the PopArt 1.7 (Leigh et al. 2015).

### ﻿Morphological analysis

Individuals of *Lathonura* were extracted from samples and placed in a glycerol-ethanol mixture drop. The dissections were performed using an Olympus SZ-51 optical binocular microscope (Olympus, Japan) following a technique described by Kotov and Štifter (2006). Line drawings were prepared with camera lucida on an Olympus CX-41 high-power optical microscope and then inked in Adobe Illustrator 26.0.3 (Adobe Systems Inc., USA) using a graphic tablet Wacom One CTL-672-N (Wacom, Japan). For taking optical micrographs, a LOMO BLM-L optical microscope with MS-12 digital camera (LOMO-MA, Russia) was used. Each specimen was photographed with gradual focus shift, with subsequent merging the frame series with Helicon Focus 8 software (Helicon Soft Ltd., Ukraine). All measurements were performed in ImageJ software (Schneider et al. 2012) using scale bars obtained from eyepiece micrometers of Olympus CX-41 and LOMO BLM-L optical microscopes.

For scanning electron microscopy, specimens were gradually dehydrated in ethanol-acetone mixture with increasing concentrations of the latter, as follows: 25%, 50%, 75%, 90%, 100%, 100% of acetone. Then the specimens were dried by a critical-point method and coated with gold. For taking microphotographs, TESCAN MIRA scanning electron microscope (TESCAN, Czech Republic) in high vacuum mode was used. Processing of micrographs and illustration combining were performed in the Adobe Photoshop CC and the Adobe Illustrator 2022 software (Adobe Systems Inc., USA). The map was created in QGIS 3.38.0 software (QGIS Development Team, USA), using Natural Earth 1:10m raster and vector data (https://www.naturalearthdata.com/downloads/). For visualizing the map, Lambert Azimuthal Equal Area projection (EPSG:3576) was applied.

In this study, terms suggested by Kotov (2013) and Fryer (1974) (with some modifications) are applied for most of the structures. For labeling parts of the thoracic limbs, the following nomenclature is used: setae of exopod are marked by lowercase Latin letters (a–d), setae of posterior endopodal row by Arabic numerals (1–13), setae of anterior endopodal row by Arabic numerals with quotation mark (e.g., 1’), posterior setae of gnathobase II–IV by Greek letters (α–δ). All setae are labeled from outer to inner limb margin. Additional setae present in male thoracic limb I are denoted by uppercase Latin letters (X, Y). It should be noted that this nomenclature does not consider setae with similar numbers (e.g., limb I seta 1 and limb II seta 1) as homologous. All other abbreviations are decoded in the respective figure captions.

The following measurements were included in the morphological descriptions:

**AL** antenna I length (without aesthetascs);

**ANL** maximal antenna II length (without swimming setae);

**APL** postabdomen anal portion length;

**ASL** basal antenna I seta length;

**AW** antenna I width, measured at base in anterior view;

**BH** maximal body height;

**BL** body length, measured as distance between supraorbital head margin and posterodorsal angle;

**BW** maximal body width;

**ED** compound eye diameter;

**ENL** endopodite length without apical spine;

**EXL** exopodite length without apical spine;

**HL** head length, measured as distance between supraorbital head margin and valve attachment in lateral view;

**HW** maximal head width;

**LL** labrum length, measured as distance from anteriormost labral keel margin to a tip of labral outgrowth;

**ML** mandible length;

**MPL** mandibular plate length;

**MPW** mandibular plate width;

**PCL** postabdominal claw length;

**PCW** postabdominal claw width, measured at claw base;

**PL** postabdomen length without claws, measured as distance from postabdominal seta base to the claw base;

**Sa, Sα, S1** length of setae a, α, and 1, respectively (length of setulae excluded);

**SM** male antenna I seta length.

## ﻿Results

### ﻿Genetic variability

Using the dg_ctd_folmer_F / HCO2198 primer pair for barcoding, we obtained a fragment ~700 bp length. However, after reviewing the resulting sequences, it became clear that in several specimens the PCR was insufficiently specific, and the obtained data did not represent the Folmer fragment of the COI gene of cladoceran origin. Using the aforementioned primer pair, only seven specimens were successfully sequenced. Hence, for the remaining specimens we replaced the reverse primer from HCO2198 to simoR, which targets a slightly larger region. In this respect, we advise against using standard Folmer-like primers for this genus, and believe that the erroneous sequence originated from nuclear or, maybe, symbiont genomes due to nonspecific annealing.

The 639 bp COI nucleotide sequences of the final alignment have a total of 202 variable sites. The best model of evolution for a maximum likelihood tree is TIM2+F+I. The final maximum likelihood tree has a likelihood score of –ln L = 2117.866.

Our results show a deep divergence between two clades of *Lathonura*: clade A, distributed in Europe and Canada (Fig. 1, blue squares), and clade B observed in East Eurasia (Fig. 1, orange circles), both with a strong bootstrap support (Fig. 2A, B). Within the clade A, three well-supported subgroups are revealed: Canadian clade A1 and European clades A2 and A3 (bootstrap support 100%, 97% and 95%, respectively, Fig. 2A). Within clade B, two subgroups are identified: B1, found in Siberia, and B2, distributed in Kamchatka (bootstrap support 81% and 100%, respectively; Fig. 2A). Uncorrected genetic distance between clusters A and B significantly exceeds the mean distances within groups (the intergroup distance is 0.22, the intragroup distances are 0.02 and 0.03, respectively; Fig. 2B).

### ﻿Morphological variability and species descriptions

The preliminary morphological and molecular analysis supports separation of the studied *Lathonura* populations into two clades (Fig. 2), based on morphology of the posteroventral valve margin. Representatives of clade A (*Lathonurarectirostris* s. l.) have uniform armature of the posteroventral valve margin (Suppl. material 1: fig. S1A–F, see also species descriptions). Populations sharing this morphological trait occur in West Eurasia (reaching the Tomsk Area of Russia in the east) and east of North America (Fig. 1, squares). In contrast, representatives of the clade B have denticles of the posteroventral valve margin differing in size (Suppl. material 1: fig. S1G–J). Populations with similar morphology are spread throughout East Eurasia (reaching Zabaikalsky Krai of Russia at the west) and also occur in Alaska, USA (Fig. 1, circles). For more information on clade morphological affinities, see the Differential diagnosis section.

Below, we provide a redescription of *Lathonurarectirostris*, mainly based on the material from Innlandet, Norway, and multiple localities in European Russia, including gamogenetic stage morphology. Also, we propose a new species name, *Lathonurabekkerae* sp. nov., for clade B (Fig. 2A, B) and populations sharing the morphological traits (Suppl. material 1: fig. S1G–J).

### ﻿Species descriptions

#### ﻿Class: Branchiopoda Latreille, 1817

**Order: Anomopoda Sars, 1865**

**Family: Macrothricidae Norman & Brady, 1867**

##### ﻿Genus: Lathonura

Taxon classificationAnimaliaAnomopodaMacrothricidae

Lilljeborg, 1853

C2DB243B-7216-5489-91B5-B3D70B8BB762

###### Type species.

*Lathonurarectirostris* (O.F. Müller, 1785).

###### Emended genus diagnosis.

**Parthenogenetic female.** Body length 0.5–1.3 mm. Body elongate, oval, slightly flattened laterally, valves with a low dorsal keel. Head lacking a rostrum, depression between head and valves weakly expressed or absent. Anterior headshield margin straight to slightly convex. A single dorsal head pore, resembling a large oval or rounded ‘fenestra’. Ventral valve margin weakly convex, bears lanceolate setae bilaterally armed by short spinulae. Gut with no loops. Abdomen lacking processes. Postabdomen small, bilobed in cross-section, with a large dorsal process bearing postabdominal setae; anal opening terminal. Preanal and anal portions are covered by transverse rows of spinulae. Postabdominal claws large, incurved, lacking basal spine; both outer and inner surfaces bear a longitudinal row of short spinulae. Antenna I long, cylindrical, ornamented by wide triangular scale-like spinulae; anterior surface bearing two scale-like serrate setae in its distal part; basal antennular seta located at a low prominence. Nine aesthetascs with acute, slightly incurved tips and subapical pores; one aesthetasc much longer than others. Antenna II long; basipodite with very short distal burrowing spine. Antenna II seta formula 0–1–1–3/1–1–3, spine formula 0–1–0–1/0–0–0. Five thoracic appendages. IDL of the limb I bearing four setae, each seta with unilateral armature of flattened setulae. Anterior setae of the limb I inner portion long, not forked. Ejector hooks small, one of them reduced to a tubercle. Limb II exopodite with one seta; inner portion with eight scraping setae in anterior row and three setae in posterior row. Limb III exopodite with four setae; distal endite with three long posterior setae and one long anterior seta. Limb IV exopodite with one seta; inner portion with three setae. Limb V with a total of four setae.

**Ephippial female.** Body length 1.1–1.2 mm. General body shape similar to that of parthenogenetic female, compressed laterally, dorsal keel low. Dorsal valve margin angulate in the anterior half, widely rounded posteriorly. Ephippium transparent to weakly melanized, ornamented by polygons or wrinkles. Two to eight eggs in the ephippium.

**Male.** Body length 0.5–0.8 mm. General body shape similar to that of young parthenogenetic female, body compressed laterally. Postabdomen shape similar to that of the female; gonopores slit-like, located subdistally on lateral surface of the postabdomen. Antenna I bearing an additional basal seta at the anteromedial side and six or seven transverse rows of more or less flattened setae along its anterior side. Antenna II basipodite with two additional distal setae at the outer side. Thoracic limb I with a prominent subdistal lobe and a hook at IDL base; anterior surface of the SDL bearing transverse rows of spinulae; posterior surface of the hook tip with three transverse cuticular ridges. IDL of the limb I and endite 3 both with one additional anterior seta.

##### ﻿ Lathonura rectirostris

Taxon classificationAnimaliaAnomopodaMacrothricidae

(O.F. Müller, 1785)

EC4A39C2-F7FB-597B-9B80-5BDB0B4BC544

Figs 3 , 4 , 5 , 6 , Suppl. materials 1 , 2: figs S1A–F, S2–4

###### References.

**Figure 4.**
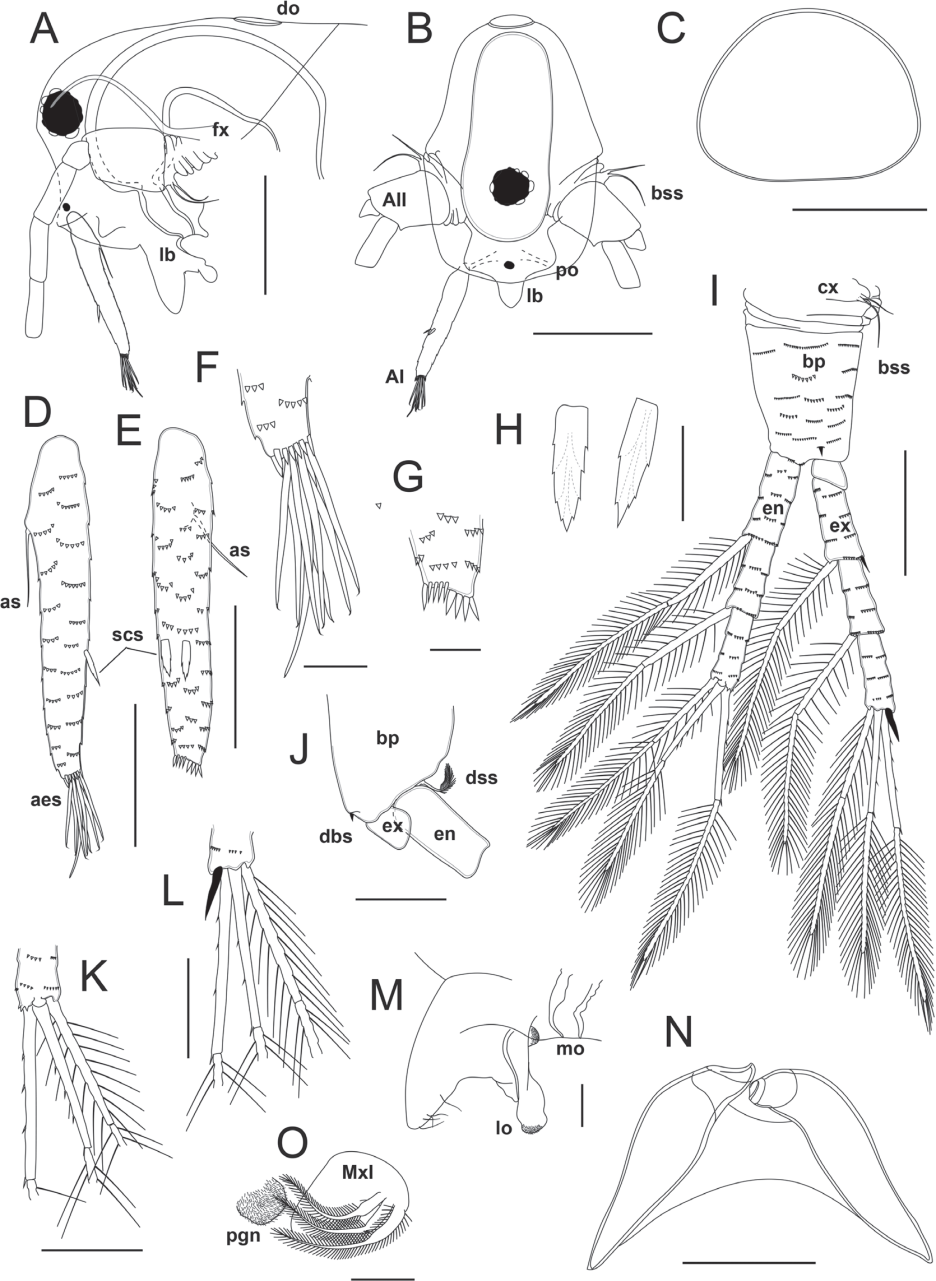
*Lathonurarectirostris* (O.F. Müller, 1785) from Lake Randsfjorden, Innlandet, Norway (Fig. 1, loc. 1), female head morphology. A. Head in lateral view (mouth appendages not shown); B. Head in anterior view; C. Dorsal organ; D–G. Antenna I: D. Lateral view; E. Anterior view; F. Tip of antenna I in posterior view; G. Tip of antenna I in anterior view; H. Scale-like setae; I–L. Antenna II; I. General outer view; J. Distal portion of basipodite, lateral view; K. Tip of endopodite; L. Tip of exopodite; M. Labrum in lateral view; N. Mandibles, general anterior view; O. Paragnath and maxilla I, ventral view. Abbreviations: AI, antenna I; AII, antenna II; aes, aesthetascs; as, basal seta of antenna I; bp, basipodite; bss, basal sensory setae; cx, coxal part of antenna II; d, dorsal molar plate side; dbs, distal burrowing spine; do, dorsal organ; dss, distal sensory seta; en, endopodite; ex, exopodite; fx, fornix; v, ventral molar plate side; lb, labrum; lo, labral outgrowth; mo, mouth opening; MxI, maxilla I; po, prelabral outgrowth; pgn, paragnath; scs, scale-like setae. Scale bars: 200 µm (A, B); 50 µm (C, J, K–N); 100 µm (D, E, I); 20 µm (F, G, H, O–Q).

**Figure 5.**
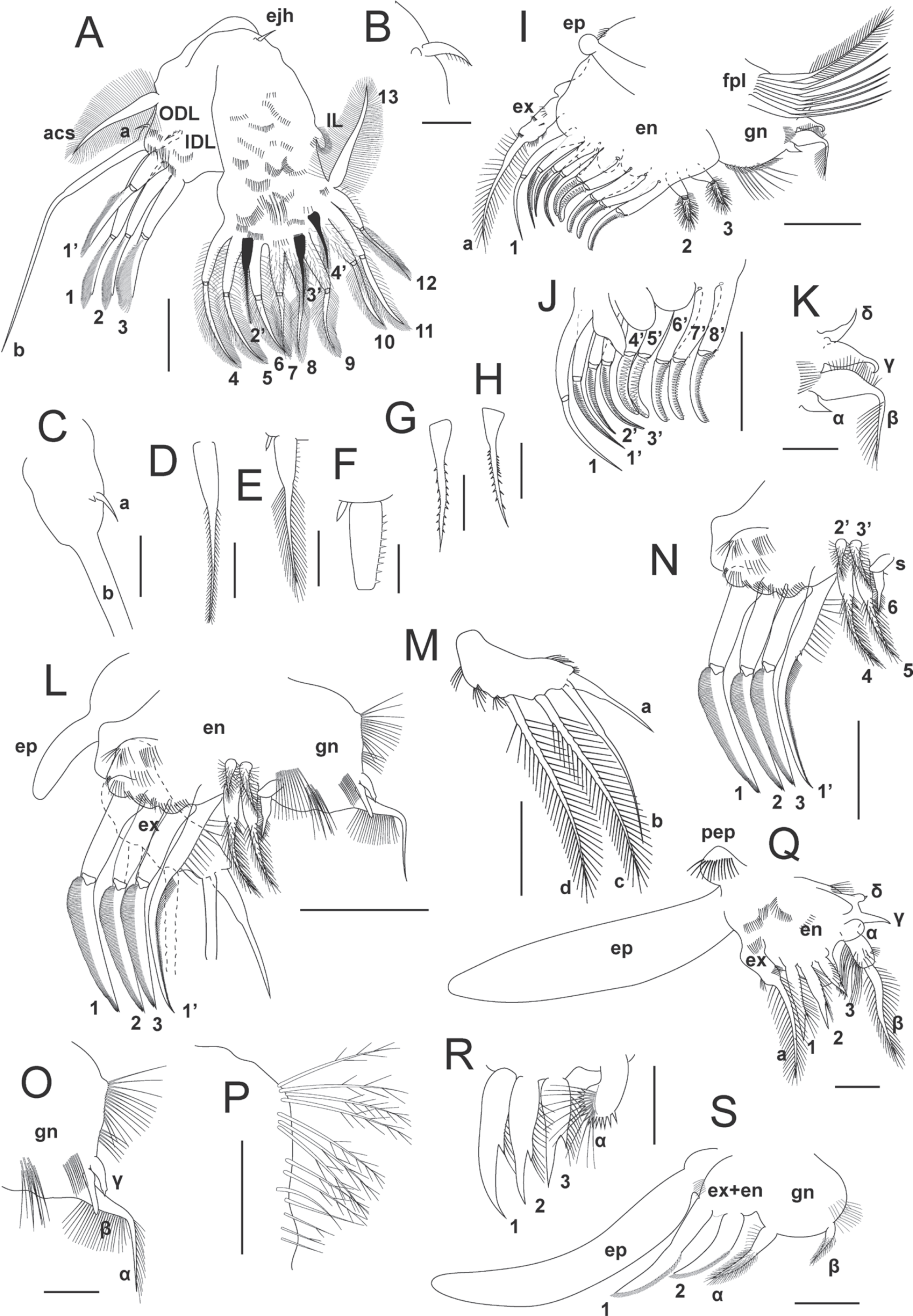
*Lathonurarectirostris* (O.F. Müller, 1785) from Lake Randsfjorden, Innlandet, Norway (Fig. 1, loc. 1), female thoracic appendages. A–H. Limb I; A. General anterior view (appendage is flattened); B. Ejector hooks; C. ODL; D. Seta 1’; E, F. Seta 2’; G, H. Seta 3’; I–K. Limb II: I. General anterior view; J. Inner limb portion, posterior view; K. Gnathobase, anterior view; L–P. Limb III; L. General inner view (note position of the exopodite); M. Exopodite, outer view; N. Inner portion, inner view; O. Gnathobase; P. Gnathobase setulae; Q, R. Limb IV: Q. General outer view; R. Inner portion; S. Limb V, outer view. Abbreviations: acs, accessory seta; gn, limb II–V gnathobase; ejh, ejector hooks; en, endopodite; ep, epipodite; ex, exopodite; pep, pre-epipodite. For seta numbering, see Methods section. Scale bars: 50 µm (A, I, J, L–N); 10 µm (B, E); 20 µm (C–E, G, H, O–S).

**Figure 6.**
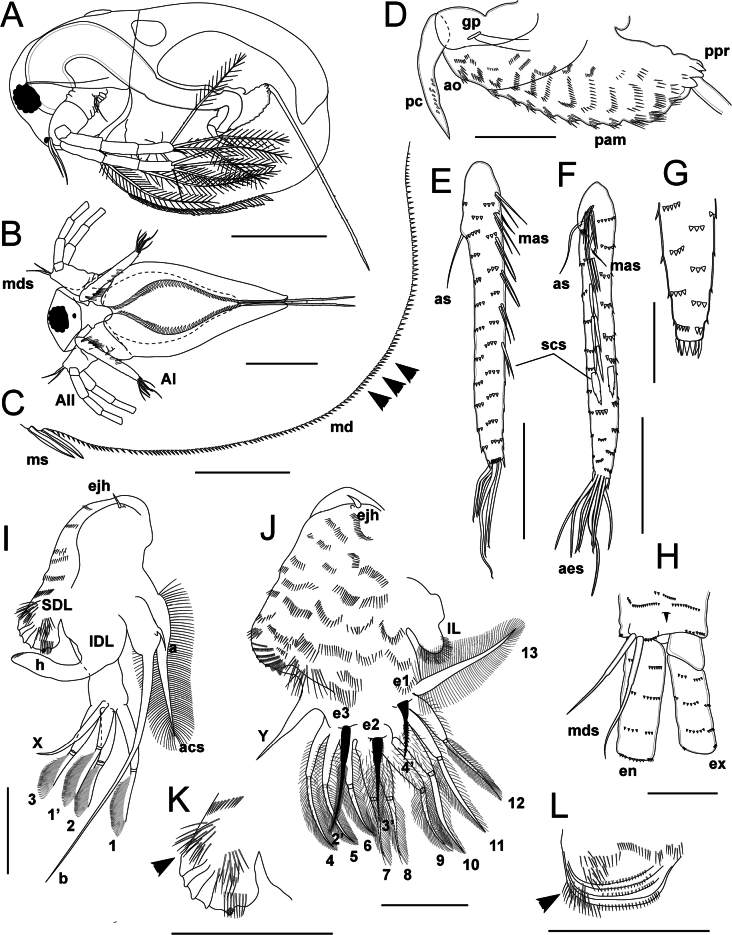
*Lathonurarectirostris* (O.F. Müller, 1785) from Lake Randsfjorden, Innlandet, Norway (Fig. 1, loc. 1), male morphology. A. General lateral view; B. Ventral view; C. Posteroventral valve margin; D. Postabdomen in lateral view; E–G. Antenna I: E. Lateral view; F. Anterior view; G. Antenna I tip in posterior view; H. Antenna II basipodite in outer view; I–L. Thoracic limb I: I. Outer view (inner endites not shown); J. Inner endites in anterior view; K. Subdistal lobe in outer view; L. Subdistal lobe in anterior view. Abbreviations: AI, antenna I; AII, antenna II; acs, accessory seta; aes, aesthetascs; ao, anal opening; as, basal seta of antenna I; gp, gonopore; ejh, ejector hooks; en, endopodite; ex, exopodite; mas, male seta of antenna I; h, IDL hook; IDL, inner distal lobe; IL, inner lobe; md, marginal denticles; mds, male distal basipodite setae; ms, marginal valve setae; pam, preanal postabdomen margin; pc, postabdominal claw; ppr, postabdominal process; scs, scale-like setae. Scale bars: 200 µm (A, B); 50 µm (C–F, H–L); 20 µm (G).

**Figure 7.**
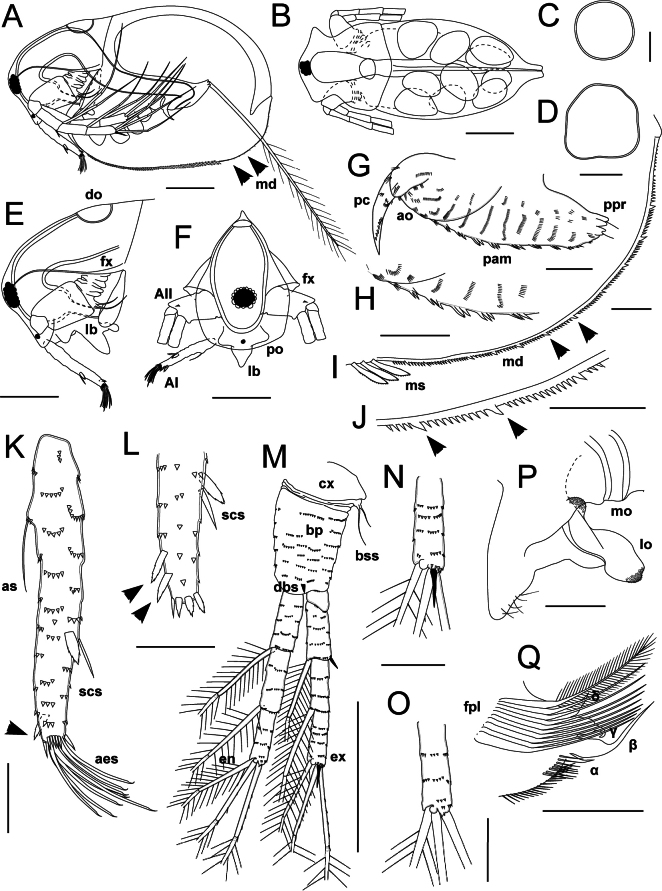
*Lathonurabekkerae* sp. nov., parthenogenetic female from a small unnamed tundra lake in Parapolsky Dol, Kamchatsky Krai, Russia (type locality, Fig. 1, loc. 31). A. General lateral view (black arrows indicate posteroventral valve armature); B. Dorsal view; C, D. Dorsal organ; E. Head in lateral view; F. Head in anterior view; G. Postabdomen in lateral view; H. Dorsal postabdomen armature; I, J. Posteroventral valve armature (arrows indicate enlarged denticles); K, L. Antenna I (black arrows indicate additional scale-like setae at posterior face of the appendage): K. Anterolateral view; L. Tip in posterolateral view; M–O. Antenna II: M. General outer view; N. Distal exopodite segment; O. Distal endopodite segment; P. Labrum in lateral view; Q. Thoracic limb II gnathobase. Abbreviations: AI, antenna I; AII, antenna II; aes, aesthetascs; ao, anal opening; as, basal seta of antenna I; bp, basipodite; bss, basal sensory setae; cx, coxal part of the antenna II; dbs, distal burrowing spine; do, dorsal organ; en, endopodite; ex, exopodite; fx, fornix; fpl, filtering plate of the limb II; lb, labrum; lo, labral outgrowth; mo, mouth opening; md, marginal denticles; ms, marginal valve setae; pam, preanal margin; pc, ppo, prelabral outgrowth; ppr, postabdominal process; scs, scale-like setae. Scale bars: 200 µm (A, B, E, F, M); 50 µm (C, G–L, N–Q).

**Figure 8.**
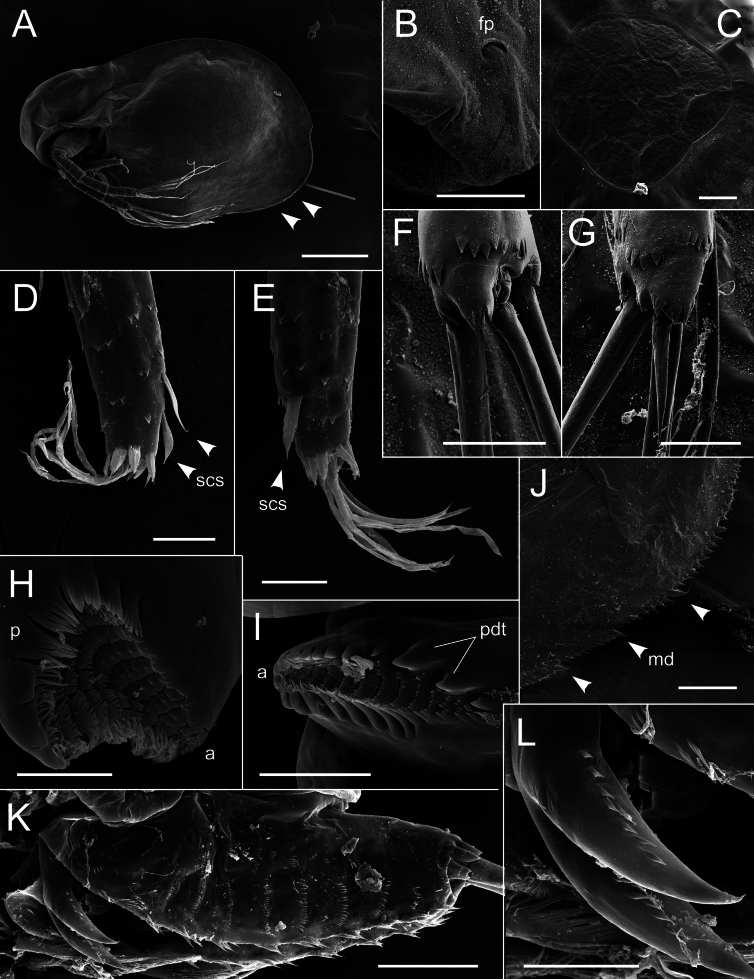
*Lathonurabekkerae* sp. nov., parthenogenetic female from a small unnamed tundra lake in Parapolsky Dol, Kamchatsky Krai, Russia (type locality, Fig. 1, loc. 31), scanning electron microscopy. A. General lateral view; B. Frontal head pore; C. Dorsal organ; D, E. Antenna I tip: D. Anterior view; E. Posterior view; F. Antenna II endopodite tip; G. Antenna II exopodite tip; H. Left mandible molar surface; I. Right mandible molar surface; J. Posteroventral valve armature; K. Postabdomen in lateral view; L. Postabdominal claws. Abbreviations: a, anterior mandible side; fp, frontal pore; md, marginal valve denticles; p, posterior mandible side; pdd, posterodorsal mandible teeth; scs, scale-like setae. White arrows indicate diagnostic features. Scale bars: 200 µm (A); 20 µm (B–G, I, J, L); 10 µm (H); 50 µm (K).

**Figure 9.**
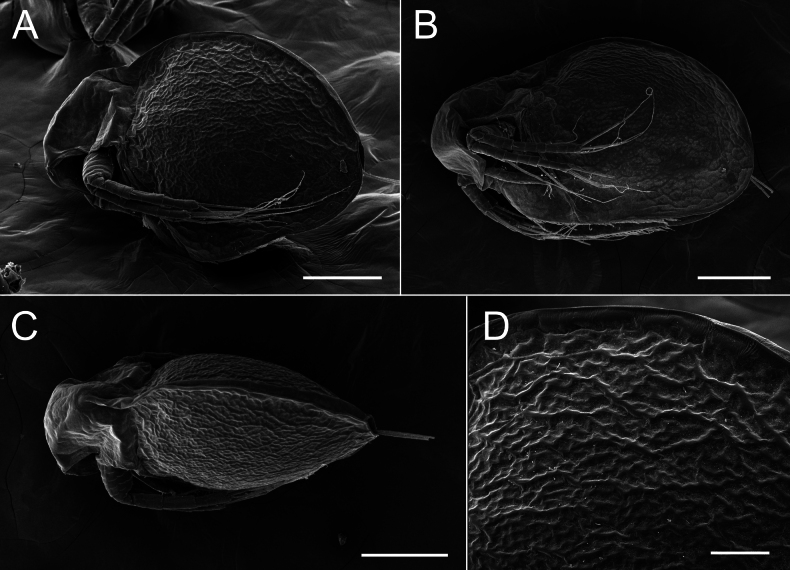
*Lathonurabekkerae* sp. nov., ephippial female from a small unnamed tundra lake in Parapolsky Dol, Kamchatsky Krai, Russia (type locality, Fig. 1, loc. 31), scanning electron microscopy. A, B. General lateral view; C. Dorsal view; D. Ephippium sculpture. Scale bars: 200 µm (A–C); 50 µm (D).

**Figure 10.**
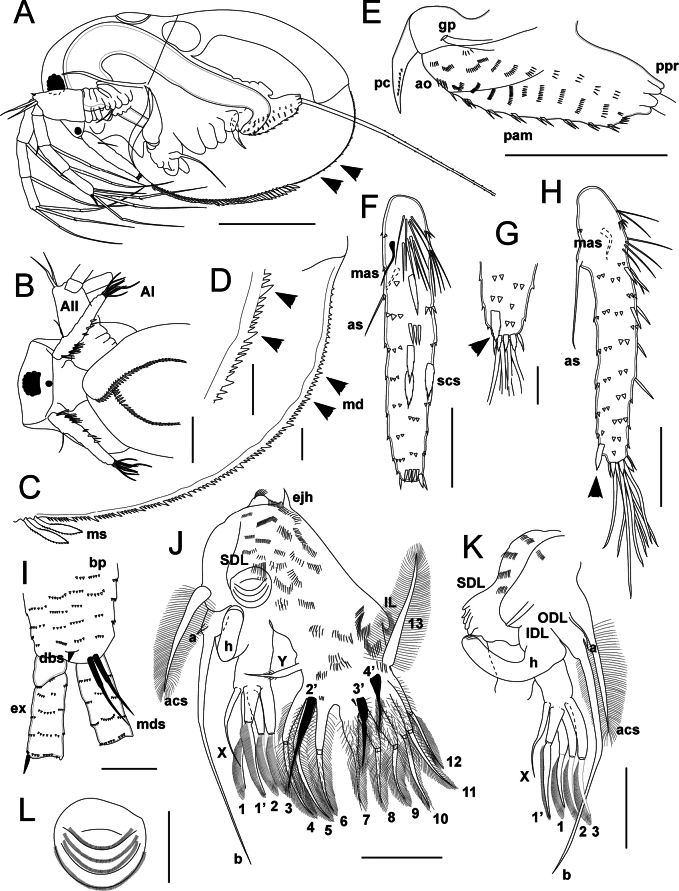
*Lathonurabekkerae* sp. nov., male from a small unnamed tundra lake in Parapolsky Dol, Kamchatsky Krai, Russia (type locality, Fig. 1, loc. 31). A. General lateral view (black arrows indicate posteroventral valve armature); B. Head in ventral view; C, D. Posteroventral valve armature (black arrows indicate enlarged denticles); E. Postabdomen in lateral view; F–H. Antenna I: F. Anterior view; G. Tip of antenna I in posterolateral view (black arrow indicates distal scale-like seta); H. Anterolateral view (black arrow indicates distal scale-like seta); I. Antenna II basipodite; J–L. Thoracic limb I: J. Anterior view; K. Outer view; L. subdistal lobe in anterior view. Abbreviations: AI, antenna I; AII, antenna II; acs, accessory seta of the limb I; ao, anal opening; as, basal seta of antenna I; bp, basipodite; dbs, distal burrowing spine; ex, exopodite; gp, gonopore; h, hook of the IDL; IDL, inner distal lobe of the limb I; IL, inner lobe; mas, male seta of antenna I; md, marginal denticles; mds, male distal basipodite setae; ms, valve marginal setae; ODL, outer distal lobe of the limb I; pam, preanal postabdomen margin; pc, postabdominal claw; ppr, postabdominal process; scs, scale-like setae; SDL, subdistal lobe of the limb I. Scale bars: 200 µm (A); 100 µm (B, E); 20 µm (C, D, J, L); 50 µm (F–K).

Müller O.F. (1785): 90, pl. XII, figs 1–3 (*Daphnia*); Koch (1841): fig. 35.24 (*Pasithea*); Zaddach (1844): 23–24 (*Daphniabrachyura*); Liévin (1848): 42, pl. XI, figs 1–3 (*Pasithea*); Fischer (1848): 174–177, pl. VI (*Daphniamystacina*); Lilljeborg (1853): 57–61, pl. IV, figs 8–11, pl. V, fig. 2; Schödler (1858): 27, pl. 1, fig. 10 (*spinosa*); Leydig (1860): 200–205, pl. VII, fig. 57 (*Pasithearectirostris*, *P.lacustris*); Müller Р.Е. (1867): 139–140; Norman and Brady (1867): 14–16, pl. XXIII, figs 8–12; Lund (1870): 155, pl. IX, figs 1–4; Gruber and Weismann (1877): 103–113, pl. IV, figs 11, 12, 14, 15, 15a (*Pasithea*); Hellich (1877): 63; Birge (1879): 89–90, Herrick (1884): 71–72, pl. D; Matile (1890): 134; Herrick and Turner (1895): 216, pl. LVII; Lilljeborg (1900): 354–360, pl. LV, figs 15–18, pl. LVI, figs 1–14; Keilhack (1909): 64, fig. 147; Birge (1918): 716, fig. 1117; Berg (1929–1930): 65–66; Behning (1941): 207–209, fig. 90; Scourfield and Harding (1958): 29, figs 62, 63; Brooks (1959): 630, fig. 27.64; Herbst (1962): 72, fig. 45a–d; Sramek-Hušek et al. (1962): 292, fig. 106; Manuilova (1964): 201–202, fig. 93; Sergeev (1971): 1–7, figs 1–6; Flössner (1972): 242–245, figs 114, 115; Fryer (1972): 80–82, figs 1–3; Fryer (1974): 211–227, figs 91–109; Smirnov (1976): 158–162, figs 143–148 (*rectirostris*, *lacustris*); Negrea (1983): 196–198, fig. 78; Margaritora (1985): 183–185, fig. 75; Dodson and Frey (1991): fig. 20.50; Dumont and Silva-Briano (1998): figs 3, 5; Silva-Briano (1998): 156–168, figs 11–19, 68–102; Flössner (2000): 79–80; Kotov (2006): fig. 6H; Hudec (2010): 258–261, fig. 62; Kotov et al. (2010): 222, pl. 128, figs 7–8, pl. 130, figs 8–10; Kotov (2013): figs 27З, 65З, 81А–Б, 100В–Г, 151E, 163A; Błedzki and Rybak (2016): 248–249; Rogers et al. (2019): figs 16.2.23L–M, Korovchinsky et al. (2021): 235–237, pl. 68, figs 10–12.

###### Type material.

Probably lost, as are all the materials of Otto Frederik Müller (Frey 1980).

###### Type locality.

Surroundings of Copenhagen, Denmark (Müller O.F. 1785).

###### Material examined.

Norway • 4 ephippial ♀, 3 ♂; Innlandet, Lake Randsfjorden; 60°47.70'N, 10°8.02'E; 17 Oct. 2015; A.Y. Sinev leg.; IEE AAK 2024–132 • 10 parthenogenetic ♀, 4 ephippial ♀, 4 ♂; same data as for preceding; 60°31.77'N, 10°17.67'E; 7 Oct. 2015; A.Y. Sinev leg.; IEE AAK M–3383. Russia • 2 parthenogenetic ♀; Murmansk Area, Teriberka; 69°11.80'N, 35°7.31'E; 4 Aug. 2020; small roadside lake with rocky sediment; P.G. Garibian leg.; IEE AAK M–5921 • 1 parthenogenetic ♀; Tver Area, Rivitsky village; 57°36.55'N, 35°54.84'E; 4 Sep. 2011; S.V. Pavlova leg.; IEE AAK M–2182 5921 • 5 parthenogenetic ♀; Moscow Area, Lake Glubokoe; 55°45.11'N, 36°30.65'E; 16 Aug. 2016; vegetated zone with water lily leaves; A.A. Kotov leg. IEE AAK M–3489 • 2 parthenogenetic ♀; Moscow Area, Lomonosov MSU biological station; 55°41.93'N, 36°43.83'E; 7 Oct. 2023; vegetated pond; E.K. Degtyareva leg.; IEE AAK M–8490 • 1 parthenogenetic ♀; Ryazan Area, Murmino; 54°34.95'N, 40°3.19'E; 15 July 2007; vegetated lake with water pineapple; A.A. Kotov leg.; IEE AAK M–0430 • 1 parthenogenetic ♀; Kostroma Area; 58°10.15'N, 44°30.57'E; 5 Sep. 2020; small vegetated pond in Unzha River valley; A.A. Neplyukhina leg.; IEE AAK M–6797 • 1 parthenogenetic ♀; Kostroma Area; 58°11.3'N, 44°33.24'E; 5 Sep. 2020; small vegetated pond in Unzha River valley; A.A. Neplyukhina leg.; IEE AAK M–6787 • 2 parthenogenetic ♀; Nizhny Novgorod Area; 56°12.69'N, 43°45.13'E; 19 Sep. 2018; Gnilichka River, vegetated shoreline with coontail; D.E. Gavrilko leg.; IEE AAK M–4471 • 5 parthenogenetic ♀; Nizhny Novgorod Area; 56°12.32'N, 43°45.41'E; 19 Sep. 2018; vegetated pond with water pineapple at Gnilichka River; D.E. Gavrilko leg.; IEE AAK M–4479 • 2 parthenogenetic ♀; Penza Area; 53°14.04'N, 45°5.28'E; 5 May 2010; vegetated oxbow lake; E.I. Bekker leg.; IEE AAK M–1808; • 2 parthenogenetic ♀; Penza Area; 53°10.36'N, 45°4.46'E; 5 May 2009; a small lake with water pineapple in the vicinity of Penza; E.I. Bekker leg.; IEE AAK M–0953 • 3 parthenogenetic ♀; Chelyabinsk Area; 55°0.68'N, 59°54.48'E; 8 Aug. 2006; a big creek on River Atlian; A.A. Kotov leg.; IEE AAK M–0325 • 2 parthenogenetic ♀; Chelyabinsk Area; 54°59.46'N, 59°50.38'E; 8 Aug. 2006; Lake Pestchanoe, Tobol River valley; A.A. Kotov leg.; IEE AAK M–0324 • 2 parthenogenetic ♀; Tomsk Area; 56°43.33'N, 84°41.52'E; 16 July 2005; a lake on right bank of Tom’ River; A.A. Kotov leg.; IEE AAK–0129 • 2 parthenogenetic ♀; Chelyabinsk Area; 56°32.63'N, 84°9.48'E; 20 July 2005; an oxbow lake near Melnikovo; A.A. Kotov leg.; IEE AAK M–0135. Mongolia • 1 parthenogenetic ♀; Uvs Aimag, Tes, Lake Uhegiin Gol; 50°29.67'N, 93°36.02'E; 30 Aug. 2006; C. Jersabek leg.; IEE AAK 2008–082. U.S.A. • 1 parthenogenetic ♀; Rhode Island, Bowdish Reservoir; 41°55.45'N, 72°13.04'W; 14 June 2004; D.J. Taylor, A.A. Kotov leg.; IEE AAK 2005–236 • 2 parthenogenetic ♀; New Hampshire, Lake Sunapee; 43°22.54'N, 73°55.80'W; 24 June 2004; W. Piel, A.A. Kotov leg.; IEE AAK 2006–165. See Suppl. material 6 for more information on localities.

###### Description.

**Parthenogenetic female.** Body length 0.5–1.3 mm. Body oval in lateral view (BL/BH = 1.5–1.6), slightly to moderately flattened laterally (BL/BW = 2.2–2.6) (Fig. 3A–C, Suppl. material 2: fig. S2A), with a well-expressed dorsal keel. Depression between head and valves is absent. Dorsal valve margin is evenly convex, maximum body height and width in the middle of the body (Fig. 3A, C, Suppl. material 2: fig. S2A). Posterodorsal valve angle weakly expressed. Posteroventral valve margin is widely rounded, bearing 90–135 uniform narrow denticles; the posteriormost 15–20 denticles might be somewhat larger than the others (Fig. 3D–G, Suppl. materials 1, 3: figs S1A–F, S3C–E). Ventral margin of intact specimen slightly convex in lateral view, slit between valves visible from ventral side expanded in its medial section (Fig. 3A, B). Ventral valve margin bears a row of 45–50 lanceolate marginal setae, slightly increasing in length posteriorly (Fig. 3D–G, Suppl. material 2: fig. S2D); the setae flattened in anteroposterior direction. Row of marginal setae reaching posterior quarter of the valve ventral margin (Fig. 3A, D–G); both inner and outer seta edges finely serrate (Fig. 3E, Suppl. material 2: fig. S2D). Anterior valve margin with a thickened cuticular flange (Fig. 3A, Suppl. material 2: fig. S2A). Carapace is almost smooth or finely reticulated (Suppl. material 2: fig. S2A), transparent to light brown, rarely dorsal body surface melanized.

Head large (HL/BL = 0.35–0.45), slightly narrowing anteriorly in dorsal view, lacking a cervical groove posteriorly. Headshield margins strongly extending laterally (HW/HL = 1.0–1.1), forming arch-shaped fornices; each fornix is concave in its medial portion in dorsal view, S-shaped in lateral view (Figs 3A, B, 4A). Dorsal head margin evenly convex in lateral view; anterior head margin concave in lateral view; anterior headshield margin straight to slightly convex in anterior view (Figs 3A, B, 4A, B). Rostrum reduced. Eye relatively small (ED/HL = 0.13–0.15), located close to dorsal head margin; ocellus small, shifted towards the anterior headshield margin (Fig. 4A, B). Ventral head margin posteriorly to rostrum evenly convex in dorsal view, roughly perpendicular to dorsal margin in lateral view (Figs 3A, 4A, Suppl. material 2: fig. S2A). Ventral head margin straight to slightly convex, bearing laterally a pair of conical prelabral outgrowths with rounded tips (Figs 3A, B, 4A, B).

Dorsal organ (= dorsal “head pore”) located at distance of 1–3× its diameter from the posterior headshield margin; approximately as large as the eye, almost rounded to oval and slightly widening posteriorly in dorsal view, smooth, sometimes weakly extending in lateral view (Figs 3A, C, 4A–C, Suppl. material 2: fig. S2B, C). Lateral head pores absent. A small horseshoe-shaped frontal pore located subterminally, close to anterior headshield margin (Suppl. material 2: fig. S2D).

Thorax long, bearing five pairs of appendages. A large, incurved midgut located in the dorsal head portion. Gut almost straight, without convolutions and caeca (Fig. 3A, C).

Abdomen strongly reduced, lacking processes, thoracic limb V closely spaced to postabdomen base (Fig. 3A).

Postabdomen small (PL/BL = 0.23–0.27), oval in lateral view, bilobed in cross-section (Fig. 3H), widening distally, with expanded postanal portion (Fig. 3I). Dorsal postabdomen margin forming a large conical process bearing postabdominal setae; apex of the process anteriorly with a semicircular row of five or six conical outgrowths surrounding postabdominal setal bases (Fig. 3H). Preanal margin 0.70–0.72× as long as the whole postabdomen, almost straight to slightly convex in lateral view, bearing groups of short spinulae. A shallow depression might present between preanal and anal margins. Anal opening terminal, shielded by expansion of postabdomen ventrally (Fig. 3I). Anal margin short, 0.25–0.26× as long as the whole postabdomen, slightly convex, bearing groups of setulae. Postabdominal claw long and thick (PCL/PL = 0.35–0.40), incurved, directed dorsally, lacking basal spine (Fig. 3H, K); base of a claw shielded by distalmost expanded postabdomen portion in lateral view (Fig. 3H). Bases of postabdominal claws closely spaced (Fig. 3I). A longitudinal row of five to nine short, triangular spinulae at outer and inner surfaces of the claw in its medial portion (Fig. 3H, I, K). Postabdominal seta 0.6–0.7× as long as the body, bi-segmented (Fig. 3A); basal segment lacking armature, distal segment with inner armature of circular cuticular thickenings, bearing four longitudinal rows of long, sparse, flattened setulae (setula length 40–60 µm, gap between the neighboring setulae in a row 25–30 µm) (Fig. 3J).

Antenna I attached closely to the anterior headshield margin, long and narrow (AL/ED = 2.9–3.0, AL/AW = 7.4–8.4), almost straight, cylindrical in cross-section, slightly narrowing distally (Fig. 4A, B, D, E, Suppl. material 2: fig. S2E, F). Basal seta of antenna I long (ASL/AL = 0.25–0.30), located at a low protuberance on the posterior side of appendage (Fig. 4D, E). Two scale-like setae located at anterior side of the antenna I in its distal half, approximately equidistant from the appendage base (Fig. 4D, E, Suppl. material 2: fig. S2F). Scale-like setae at ventral margin triangular, flattened, with serrated margins (Fig. 4H, Suppl. material 2: fig. S2G). Tip of antenna I armed by a semicircular row of spinulae similar in shape to the scale-like setae but much smaller; a row of five to eight thin spinulae located anteriorly at the tip of the antenna I (Fig. 4F, G, Suppl. material 2: fig. S2E, F). Nine terminal aesthetascs, posteriormost one 1.3–1.4× longer and thicker than the others (Fig. 4F, G); aesthetasc pores located subterminally, tip of each aesthetasc acute, hook-shaped (Suppl. material 2: fig. S2H). The whole antenna I surface is covered by transverse rows of 3–7 wide and short flattened triangular spinulae (Fig. 4D, E, Suppl. material 2: fig. S2E, F).

Antenna II large (ANL/BL = 0.40–0.50). Coxal portion folded, slightly flattened in anteroposterior direction, bearing two sensory setae subdistally. Basal sensory setae located at low conical outgrowth, one of the setae 0.65–0.85× as long as the other (Fig. 4I, Suppl. material 2: fig. S2J). Basipodite 0.3× as long as the antenna II, subconical; outer surface of basipodite covered by transverse rows of short flattened triangular spinulae (Fig. 4I, Suppl. material 2: fig. S2J). Outer side of basipodite bearing distally a very short distal burrowing spine, ~0.1× as long as basipodite itself (Fig. 4I, J, Suppl. material 2: fig. S2I). Inner side of basipodite bearing a distal sensory seta, 5× as long as the spine (Fig. 4J); distal portion of the seta with feather-like armature of fine setulae. Antenna II branches are covered by transverse rows of wide triangular flattened spinulae (Fig. 4I, Suppl. material 2: fig. S2K, M). Exopodite four-segmented, slightly longer than the endopodite (EXL/ENL = 1.1); basal exopodal segment shortened; the segments 2–4 subequal in length, each 0.3× as long as the exopodite. Endopodite three-segmented, segment 1 is the longest, 0.35–0.37× as long as the branch; segments 2 and 3 subequal in length, 0.31–0.33× as long as exopodite (Fig. 4I); the endopodal segment 3 apically with a small, rounded lobe, visible in outer view, and an anterior row of spinulae (Fig. 4K).

Antenna II seta formula 0–1–1–3/1–1–3 (Fig. 4I, K, L, Suppl. material 2: fig. S2K, M). All the swimming setae bi-segmented, with apical segment flattened dorsoventrally. Setae of basal exopodal and endopodal segments subequal in length, 0.9× as long as the exopodite; basal and distal setal portions with a bilateral armature of uniform, long, flattened setulae (20–30 µm length, gap between neighboring setulae ~6 µm, Suppl. material 2: fig. S2L). Apical setae are similar in both branches: the posteriormost seta is the shortest, 0.9× as long as the exopodite; seta structure similar to the setae described above (Fig. 4K, L, Suppl. material 2: fig. S2K, M). Two anterior setae of similar structure: basal portion lacking setae, bearing 2–5 spinulae along the posterior margin; distal portion setulated, similar to that of the remaining setae (Fig. 4K, L, Suppl. material 2: fig. S2K, M). The anteriormost apical seta is the longest, 1.2× as long as medial apical seta; the medial seta is as long as the posterior apical seta (Fig. 4I). Spine formula 0–1–0–1/0–0–0 (Fig. 4I, Suppl. material 2: fig. S2I). The spine of exopod segment 2 is relatively short, 0.25–0.27× as long as the corresponding segment; the apical exopodal spine is 0.45–0.50× as long as the corresponding segment (Fig. 4I, L).

Labrum relatively small (LL/ED = 1.5–1.6), lacking a labral keel; anterior labral portion triangular, with rounded tip; posterior side of labrum tip l bearing few long setulae. Distal labral outgrowth rounded, finely setulated (Fig. 4M). A pair of small rounded paragnaths preceding mouth opening (Fig. 4M, O), bearing fine setulae.

Mandible small (ML/HL = 0.20–0.25). Two mandibles asymmetrical (Fig. 4N, Suppl. material 3: fig. S3A, B). Right mandibular molar plate oval (MPL/MPW = 2.2), slightly expanding posteriorly (Suppl. material 3: fig. S3A); dorsal margin of the right molar plate convex, bearing six or seven thin spines anteriorly and three robust teeth posteriorly; the teeth decrease in size posteriorly (Suppl. material 3: fig. S3A). Ventral margin slightly sinuous, bearing eight to ten transverse ribs anteriorly (Suppl. material 3: fig. S3A). Medial molar plate portion formed by 10–12 diagonal flattened outgrowths ornamented by tubercles, dense and prominent at anterior mandible edge and becoming lower and sparser towards posterior (Suppl. material 3: fig. S3A). Left mandibular molar plate subtriangular (MPL/MPW = 2.3), posterior end wider than the anterior one (Suppl. material 3: fig. S3B). Left molar plate dorsal margin convex, lacking outgrowths, ventral margin slightly concave, armed by 12–13 ribs. Medial molar plate portion armed by nine or ten diagonal rows of subrectangular outgrowths, the ventralmost outgrowths bearing digitate processes becoming longer towards posterior (Suppl. material 3: fig. S3B). The posterior edge is almost straight, armed by long digitate processes (Suppl. material 3: fig. S3B).

Maxilla I small, spatulate, incurved towards the mouth opening, bearing three bi-segmented setae densely covered by long setulae from base to tip (Fig. 4Q).

Maxilla II absent.

Five pairs of thoracic limbs, differing in size and structure (Fig. 5, see also Suppl. material 5: fig. S5).

Limb I is the largest, bent in half, consisting of outer distal lobe (ODL), inner distal lobe (IDL), three inner endites and inner lobe (IL) (Fig. 5A). Long thick accessory seta on outer side of limb, basally to the ODL; accessory seta densely covered by long setulae from base to tip (Fig. 5A). ODL short, cylindrical, bearing two setae (a, b), seta a very short, naked, located subapically; a short spine might present at base of the seta (Fig. 5C). Seta b located apically, thick, 2.6–2.7× as long as the accessory seta, naked; seta b bent medially, basal and distal portions of the seta almost straight (Fig. 5A, C). IDL short and wide, bearing four apical setae (1–3, 1’). Posterior setae (1–3) of similar structure, bi-segmented, with apical portion approximately as long as the basal one, the apical portion bearing a row of closely spaced long flattened spinulae with hook-like tips (Fig. 5A). Setae 1–3 slightly decreasing in length, seta 1 the longest (S1/Sb = 0.60–0.65). Anterior seta (1’) similar to that of posterior row but shorter (S1’/S1 = 0.70–0.75, S1’/S3 = 0.80–0.85), with apical segment exceeding the basal one (Fig. 5A). Endite 3 bearing three posterior setae (4–6) and one anterior seta (2’) (Fig. 5A). Setae 4 and 5 similar in length (S4/S1 = 0.85–0.90), seta 5 slightly shorter. All three setae bi-segmented; basal portion bilaterally armed by long setulae, apical portion bearing similar armature and additional row of 4–10 setulae at anterior surface (Fig. 5A). Anterior seta (2’) relatively long (S2’/S4 = 0.6), wide at base and narrowing distally, bilaterally armed by short setulae (Fig. 5A, D). Endite 2 bearing three posterior setae (7–9) and one anterior seta (3’). Setae 7–9 similar in size and structure (S7/S4 = 0.70–0.75), bi-segmented; basal portion densely covered by spinulae, apical portion similar to that of the setae 4–6 (Fig. 5A). Seta 3’ relatively long, wide at base and narrowing distally; basal portion bearing a row of 6–12 setulae of spines along the inner margin, apical portion bilaterally armed by long closely spaced setulae (Fig. 5A, E); a short spine located near the base of the seta laterally (Fig. 5E, F). Endite 1 bearing three bi-segmented posterior setae (10–12), a single unsegmented posterior seta (13) and a single anterior seta (4’) (Fig. 5A). Seta 10 slightly shorter than seta 11 (S10/S11 = 0.80–0.85), equal in length to this in setae 7–9; seta 10 basal portion covered by dense setulae, distal portion bearing bilateral armature of long setulae; the setulae of outer row are more closely spaced than that of inner row. Seta 11 with a basal portion bilaterally armed by long setulae; seta 11 distal portion similar to that of the seta 10. Seta 12 slightly shorter than the seta 10 (S12/S10 = 0.90–0.95), bi-segmented; seta 12 basal portion with bilateral armature of long sparse setulae, distal portion with two rows of closely spaced long setulae (Fig. 5A). Seta 13 is similar in length and structure to accessory seta (Fig. 5A). Anterior seta (4’) relatively short (S4’/S3’ = 0.85), bearing two rows of 8–15 short thick spinulae (Fig. 5G, H). Inner lobe is short, densely covered by long setulae. Two ejector hooks, first hook short, slightly incurved, armed by row of sparse setulae, second hook reduced to a tubercle (Fig. 5A, B). Anterior limb surface is covered by transverse rows of spinulae; base of endite 2 bearing a cluster of long setulae (Fig. 5A).

Limb II consists of a small rounded epipodite, cylindrical exopodite and wide inner portion (Fig. 5I). Exopodite bearing a single seta (a), long to relatively short (Sa/S1 = 0.45–0.80) wide at base and progressively narrowing distally, armed by two rows of long sparse setulae. Posterior side of exopodite armed by two groups of spinulae; exopodite apex bearing a transverse row of spinulae anteriorly, at base of the seta a. Inner portion bearing three posterior setae (1–3) and eight anterior scraping setae (1’–8’) (Fig. 5I, J). Scraper 1’ is the longest (S1’/S1 = 1.2), scraper 2’ is shorter than scrapers 1’ and 3’ (S2’/S1 = 0.80–0.85, S2’/S3’ = 0.9), scraper 4’ is the shortest (S4’/S1’ = 0.75–0.77, scrapers 5’–8’ progressively increase in length towards the gnathobase (S5’/S1’ = 0.8–0.82, S8’/S1’ = 0.93–0.95). Scrapers 1’–3’ armed by a row of thin spinulae; scrapers 4’ and 5’ with 15–20 thick spines; scrapers 5’–8’ with 25 or more relatively thin spines. Seta 1 located behind the scraper 1’ attachment, exceeds length of this scraper; seta 1 bi-segmented, naked, slightly incurved. Three lobes of different shape located behind bases of scrapers 1’–6’: outermost lobe long and narrow, expanding medially, with acute apex; medial lobe relatively short, oval; innermost lobe short and wide (Fig. 5J). Setae 2 and 3 shifted towards the gnathobase, short, bi-segmented; distal portion of each seta bearing dense short setulae (Fig. 5I, J). Gnathobase large, lobate, bearing four posterior elements (α–δ): a very short naked seta (α), a long bi-segmented seta (β), bent medially; the seta β basal portion inflated, distal portion thin, both bearing row of long setulae; hook-shaped short seta with inflated base (γ); very short naked seta (δ), slightly longer than seta α (Fig. 5I, K). Filtering comb consisting of seven or eight long bi-segmented setae, similar in length and structure, with distal portion armed by two rows of long closely spaced setulae (Fig. 5I). Outer gnathobase margin bearing a row of numerous short closely spaced setulae and 7–12 long setulae (Fig. 5I).

Limb III is large, bent in half, consisting of short oval epipodite, elongate exopodite, and wide inner portion (Fig. 5L–P). Exopodite flattened, widening distally, slightly incurved, bearing four unsegmented setae (a–d): setae a and b short (Sa/Sc = 0.47–0.50, Sb/Sc = 0.85–0.88), naked, wide at base, with narrow distal portion; setae c and d equal in length, progressively narrowing distally, bearing two rows of long sparse setulae from base to tip (Fig. 5M). Several transverse rows of long setulae at base of the exopodite (Fig. 5M). Inner portion distal endite bearing three posterior setae (1–3) and one anterior seta (1’). The setae 1–3 subequal in length, long (S1/EXL = 1.6–1.7), bi-segmented, with distal portion bearing a row of closely spaced flattened spinulae (Fig. 5L, N). Anterior seta (1’) roughly equal in length to the setae 1–3, unsegmented, slightly incurved, with basal portion bearing two rows of long sparse setulae; seta 1’ distal portion bearing a row of short closely spaced setulae (Fig. 5L, N). Inner endite bearing three unsegmented posterior setae (4–6) and two unsegmented anterior setae (2’ and 3’). Setae 4 and 5 similar in length and structure, relatively long (S1/S4 = 0.45–0.48), with distal portion bearing dense short setulae; the seta 6 short (S6/S4 = 0.40–0.45), bottle-shaped, with distal portion bearing short setulae (Fig. 5L, N). Setae 2’ and 3’ are similar in length and structure, short (S2’/S4 = 0.35–0.45), armed by dense setulae from base to tip (Fig. 5L, N). A short oval sensillum at base of the seta 6, almost equal in length to the seta (Fig. 5L, N). Gnathobase with three elements: a long seta bent medially (α) and two cylindrical sensillae with acute tips (β, γ) (Fig. 5L, O). Inner portion distal endite covered by transverse rows of long setulae; two clusters of long setulae located at inner portion closely to the distal sensillum; basal portion of the gnathobase with 12–20 long setulae armed by secondary processes (Fig. 5O, P).

Limb IV is small, consisting of a short setulated pre-epipodite, long epipodite, short cylindrical exopodite and wide inner portion (Fig. 5Q). Exopodite bearing a single unsegmented seta (a), armed by two rows of long sparse setulae (Fig. 5Q). Inner portion of distal endite with three unsegmented setae (1–3), progressively decreasing in size basally (S1/Sa = 0.8, S2/Sa = 0.7, S3/Sa = 0.5); basal portion of each seta bearing a row of setulae along the inner margin, ending with a short thick spine (Fig. 5R). Gnathobase with four elements (α–δ) (Fig. 5Q). Distalmost element (α) lobate, expanding distally, with distal margin armed by row of spinulae; a bunch of long setulae at anterior surface of lobe (Fig. 5R). Seta β long, bi-segmented, with basal portion inflated, bearing row of long setulae along the outer margin; distal portion with two rows of long sparse setulae (Fig. 5Q). Proximal elements (γ and δ) short, γ longer than δ (Fig. 5Q). Exopodite and inner portion with several transverse rows of setulae; cluster of setulae at base of gnathobase.

Limb V is as large as limb IV, consists of large epipodite and relatively small inner portion (Fig. 5S). Exopodite and endopodite merged, bearing two thick unsegmented setae (1 and 2) of similar structure; the seta 1 1.4× as long as the seta 2, both setae slightly incurved, with basal portion naked, distal portion bearing several closely spaced longitudinal rows of short spinulae along the posterior margin; (Fig. 5S). Gnathobase relatively small, rounded, bearing two setae (α and β), the seta α 3× longer than the seta β, both seta distal portions densely covered by short setulae, seta α basal portion bearing a row of long setulae along the outer margin (Fig. 5S). Gnathobase with two clusters of setulae basally (Fig. 5S).

**Ephippial female.** Body length 1.0–1.2 mm. General morphology is very similar to that of parthenogenetic females (Fig. 3L, M, Suppl. material 3: fig. S3F, G). Body high, moderately flattened laterally (BH/BL = 0.65–0.70, BW/BL = 0.30–0.35). Dorsal valve margin forming an angle in its anterior half, dorsal keel low (Fig. 3L, Suppl. material 3: fig. S3F). Ephippium thin, transparent to light brown, weakly melanized, containing two to eight eggs. The ephippium uniformly ornamented by well-defined irregular polygons (Fig. 3L, M, Suppl. material 3: fig. S3F–H). Ephippium surface inside the polygons is almost smooth, at most bearing shallow pits (Suppl. material 3: fig. S3H). Morphology of head and thoracic appendages similar to that of parthenogenetic females.

**Male.** Body length 0.5–0.8 mm. General body shape similar to that of parthenogenetic female, body compressed laterally (BH/BL = 0.63–0.67, BW/BL = 0.40–0.42) (Fig. 6A, B). Ventral and posteroventral valve armature similar to that of parthenogenetic female (Fig. 6C).

Head, thorax, and abdomen as in parthenogenetic females (Fig. 6A, B).

Postabdomen is weakly modified in comparison to that of parthenogenetic female (PL/BL = 0.22–0.25) (Fig. 6D, Suppl. material 4: fig. S4A). Postabdominal claw long and thick (PCL/PL = 0.45–0.48), incurved, directed dorsally (Fig. 6D). Gonopore opening subdistally at lateral surface of the postabdomen; gonopore small, slit-like (Fig. 6D, Suppl. material 4: fig. S4B).

Antenna I long and slender (AL/ED = 4.28–4.30, AL/AW = 0.12–0.15), cylindrical in cross-section (Fig. 6E, F, Suppl. material 4: fig. S4C, D). Basal seta of antenna I 0.18–0.20× as long as the antenna I, seta located posteriorly at low expansion (Fig. 6E, Suppl. material 4: fig. S4C). Additional male seta (mas) located at anterior side of appendage, male seta more distant from the antenna I base than basal antennular seta (Fig. 6E, F), 0.3–0.4× as long as basal seta. Anterior surface of antenna I bearing six additional clusters of modified setae, becoming wider and more flattened towards the antenna I apex (Fig. 6E, F, Suppl. material 4: fig. S4C–E, arrows): first group consists of six to eight closely spaced thin setae of similar structure, the next include two or three flattened setae forming a transverse row. A pair of scale-like setae (scs) in distal half of the antenna I, the setae similar in shape and position to that of parthenogenetic female (Fig. 6E, F, Suppl. material 4: fig. S4C). Nine terminal aesthetascs as in parthenogenetic females, the longest one 0.37–0.39× as long as the whole antenna, 1.2–1.5× as long as the other aesthetascs (Fig. 6E, F, Suppl. material 4: fig. S4C). Antenna I sculpture is similar to that of a parthenogenetic female (Fig. 6E–G, Suppl. material 4: fig. S4C–E).

Antenna II in general is similar to that of parthenogenetic female (ANL/BL = 0.50–0.55) (Fig. 6A). Two additional setae (mds) located at distal end of the basipodite, between exopodite and endopodite attachments (Fig. 6H, Suppl. material 4: fig. S4F, G); the anterior seta is 0.80–0.90× as long as the posterior one; both setae naked (Fig. 6H, Suppl. material 4: fig. S4F, G).

Mouth parts as in parthenogenetic females.

Thoracic limb I is highly modified, consisting of outer distal lobe (ODL), inner distal lobe (IDL), subdistal lobe (SDL), inner endites, and small inner lobe (IL) (Fig. 6I, J, Suppl. material 4: fig. S4H–M). Accessory seta and ODL morphology similar to that of parthenogenetic female. IDL bearing four apical setae (1–3, 1’) similar to that of parthenogenetic female (Suppl. material 4: fig. S4J, K) and additional seta (X) located anteriorly to the seta 1’. Seta X approximately as long as the seta 1’ (SX/S1’ = 0.90–0.95), slightly incurved in its distal portion, naked (Fig. 6I, Suppl. material 4: fig. S4J). Large melanized hook present at the IDL basal portion outer side (Fig. 6I, Suppl. material 4: fig. S4H, I); the hook moderately incurved, with slightly narrowed tip armed by three transverse cuticular ribs (Suppl. material 4: fig. S4H, M). SDL large, located at anterior face of the limb proximally to the IDL attachment; SDL approximately reaching distal hook portion, rounded, armed by 5–6 transverse rows of minute closely spaced spinulae (Fig. 6K, L, Suppl. material 4: fig. S4L, M); four groups of long setulae at outer surface of the SDL (Fig. 6K, L, Suppl. material 4: fig. S4I, L, arrows). General morphology of inner endites similar to that of parthenogenetic female (Fig. 6J). Endite 3 outer side bearing an additional seta (Y) directed towards the IDL (Fig. 6J); seta Y relatively short (SY/S4 = 0.60–0.65), with basal portion progressively narrowing distally and narrow distal portion, naked (Fig. 6J). Ejector hook morphology similar to that of parthenogenetic female. Anterior limb face with transverse rows of setulae; a group of long setulae located proximally to the SDL (Fig. 6J).

Limbs II–V similar to that of parthenogenetic females.

###### Distribution and ecology.

The known range of *Lathonurarectirostris* s. str. includes the whole territory of Europe except continental Portugal, Spain, and the Mediterranean islands, although it is found in Sicily (Błedzki and Rybak 2016), European Russia (Kotov et al. 2010; Korovchinsky et al. 2021), and West Siberia east to the Tomsk Area of Russia (i.e., predominantly east of the Ob’ River). The species is, most probably, rather common in North Eurasia (although rarely recorded in zooplankton monitoring studies due to ecological affinities), but the southern distribution borders of the species remain unclear. *Lathonurarectirostris* s. l. was also observed in North Canada and eastern part of the USA: Minnesota (Herrick 1884; Herrick and Turner 1895) and Wisconsin (Birge 1892, see also Smith 2001). Parthenogenetic females of at least some East Nearctic populations share diagnostic characters with *L.rectirostris* s. str. (Suppl. material 1: fig. S1F) but genetic data show they belong to a deeply divergent clade and might represent another species (Fig. 2A, B, clade A1).

*Lathonurarectirostris* s. str. prefers weakly acidic waters with pH 5.5–7.5 (Sergeev 1971; Fryer 1974; Błedzki and Rybak 2016). The species inhabits a variety of vegetated water bodies with sandy or stony sediments and organic debris (Błedzki and Rybak 2016). *Lathonurarectirostris* is able to swim but spends most of the time attached to the substrate by setae of limb I IDLs (Fryer 1974). For slow movement along the substrate, limbs I and III are used (Sergeev 1971; Fryer 1974) instead of pushing by means of the postabdomen or antenna II typical for most macrothricids (Fryer 1974). *Lathonurarectirostris* consumes a variety of epibiont algae scraped from the aquatic plant leaves or other substrates (Błedzki and Rybak 2016). The ephippial female attaches the ephippium to submerged plants with a sticky secretion (Fryer 1974).

##### ﻿ Lathonura bekkerae sp. nov.

Taxon classificationAnimaliaAnomopodaMacrothricidae

10C4A3B3-34DF-500C-BCBC-B9AA17DF8875

https://zoobank.org/C65C2137-6638-409D-8569-4447C5932F7D

Figs 7 , 8 , 9 , 10 , Suppl. materials 1 , 5: figs S1G–J, S5

###### References.

Du (1973): 59, fig. 45A, B; Smirnov (1992): 109–111, fig. 469–474 (*rectirostris*); Dadykin et al. (2025): fig. S2 (cf.rectirostris).

###### Type locality.

Russia, Kamchatsky Krai, Parapolsky Dol, a small unnamed tundra lake (61°17.58'N, 164°42.30'E; Fig. 1, Suppl. material 6, loc. 31)

###### Type material.

***Holotype***: Russia • 1 parthenogenetic ♀; Kamchatsky Krai, Parapolsky Dol; 61°17.58'N, 164°42.30'E; 5 Sep. 2017; small tundra lake; E.I. Bekker leg.; access number MGU MI 285; original number IEE AAK M–5887. ***Paratypes*.** Russia • 11 parthenogenetic ♀; access number MGU MI 286; as for holotype • 19 parthenogenetic females; as for holotype; access number IEE AAK 2025–007.

###### Other material examined.

Russia • 2 parthenogenetic ♀; Krasnoyarsky Krai, Severtsov IEE biological station “Mirnoe”; 62°21.24'N, 89°2.49'E; 17 July 2011; oxbow lake; A.A. Kotov leg.; IEE AAK M–2146 • 1 parthenogenetic ♀; Irkutsk Area, Bazhei, Iret’ River; 52°58.28'N, 102°38.81'E; 18 Aug. 2019; A.A. Kotov, D.P. Karabanov leg.; IEE AAK M–5109 • 1 parthenogenetic ♀; Irkutsk Area, Angarsk, Bolshoi Kanal River; 52°29.06'N, 103°57.22'E; 18 Aug. 2019; A.A. Kotov, D.P. Karabanov leg.; IEE AAK M–5172 • 3 parthenogenetic ♀; Irkutsk Area; 52°1.64'N, 105°24.76'E; 16 Aug. 2023; small vegetated lake; I.A. Dadykin, A.A. Kotov leg.; IEE AAK M–8189 • 4 parthenogenetic ♀; Zabaikalsky Krai, Chasovinka, Shilka River; 53°29.20'N, 120°3.04'E; 24 Aug. 2018; D.P. Karabanov, A.A. Zharov, M.A. Gololobova, A.A. Kotov leg.; IEE AAK M–5000 • 10 parthenogenetic ♀, 1 ephippial female; Yakutia, Maragas; 62°12.28'N, 127°44.84'E; 19 June 2022; roadside puddle; L.V. Andreeva, P.G. Garibian leg.; IEE AAK M–7136 • 5 parthenogenetic ♀; Yakutia, Yakutsk; 62°1.11'N, 129°45.42'E; 5 Sep. 2011; lake; E.I Bekker, A.I. Klimovski leg.; IEE AAK M–2292 • 1 parthenogenetic ♀; Yakutia, Tuluna; 62°48.36'N, 131°4.50'E; 10 Sep 2011; E.I Bekker and A.I. Klimovski leg.; IEE AAK M–2315 • 1 parthenogenetic ♀; Magadan Area, Snezhny, Lake Dukga; 59°44.37'N, 150°51.36'E; 12 June 2016; N.G. Sheveleva leg.; IEE AAK M–6502; • 6 parthenogenetic ♀; Kamchatsky Krai, Parapolsky Dol; 61°22.62'N, 164°44.28'E; 5 Sep. 2017; small tundra lake; E.I. Bekker leg.; IEE AAK M–5890 • 2 parthenogenetic ♀; Kamchatsky Krai, Karaginsky Island; 58°52.58'N, 163°48.55'E; 6 Aug. 2022; vegetated ditch in peat lake; I.A. Dadykin leg.; IEE AAK M–7722. U.S.A. • 2 parthenogenetic ♀; Alaska, Seward Peninsula; 65°5.34'N, 165°4.63'W; 4 Aug 2011; tundra lake in Quatrine River basin, Seward Peninsula; A.A. Kotov and D.J. Taylor leg.; AAK M-6657.

###### Diagnosis.

Body length of parthenogenetic female 0.5–1.2 mm, dorsal keel well developed. Ventral valve margin with flattened setae typical for the genus. Posteroventral valve margin armed by groups of short thin denticles alternating with solitary larger denticles. The anterior headshield margin is slightly convex. Dorsal organ large, rounded, ornamented by a net-like sculpture. Antenna I distal end with one or two additional scale-like setae at the posterior side. Ephippial female with angulate dorsal margin and low dorsal keel. Ephippium ornamented by wrinkles, not forming regular polygonal sculpture. Subdistal lobe of male thoracic limb I lacking groups of spinulae at outer surface.

###### Description.

**Parthenogenetic female.** Body length 0.5–1.2 mm. Body oval in lateral view, slightly to moderately compressed laterally (BL/BW = 1.6–1.7, BL/BH = 0.35–0.50) (Figs 7A, B, 8A). Body with a low dorsal keel originating posteriorly to headshield margin (Fig. 7B). Ventral valve armature typical for the genus (Fig. 7A, H). Posteroventral valve margin bearing clusters of 5–12 short thin denticles alternating with solitary (very rarely double) longer and thicker denticles (10–15 denticles per valve); valve margin closely to posterodorsal angle usually lacking large denticles (Figs 7I, J, 8J, Suppl. material 1: fig. S1G–J). Carapace light brown, almost smooth, lacking reticulation (Fig. 8A).

Head typical for the genus (HL/BL = 0.30–0.35, HW/HL = 0.9–1.0) (Fig. 7D, E). Dorsal organ rounded, with net-like ornamentation formed by cells margins (Figs 7C, D, 8C). Frontal head pore horseshoe-shaped (Fig. 8B). Anterior headshield margin slightly convex (Fig. 7F). A pair of conical prelabral outgrowths at lateral head surface (Fig. 7E, F).

Thorax and abdomen typical for the genus. Gut straight, lacking convolutions (Fig. 7A).

Postabdomen typical for the genus (PL/BL = 0.18–0.22), with dorsal margin bearing transverse rows of five to seven spinulae; lateral postabdomen surface covered by groups of short thin spinulae (Figs 7G, H, 8K). Postabdominal claw long and thick (PCL/PL = 0.35–0.40, PCL/PCW = 4.7–4.8), slightly incurved, bearing a longitudinal row of six to ten short spinulae at both outer and inner margin (Figs 7G, 8L). Postabdominal seta typical for the genus (Fig. 7A).

Antenna I is long and narrow (AL/ED = 3.0–3.1, AL/AW = 6.9–7.0), cylindrical in cross-section, almost straight in anterior view (Fig. 7A, E, F, K, L). The basal seta of the antenna I located at low expansion, the seta 0.20–0.25× as long as the whole antenna I (Fig. 7K). Two scale-like setae located at distal half of the antenna I at its anterior face; the seta morphology is typical for the genus (Fig. 7K, L). One or two shorter scale-like setae located at the posterior face of the antenna I close to its distal end (Fig. 7L, black arrow; Fig. 8D, E, white arrows). Nine aesthetascs typical for the genus (Figs 7K, 8E). A distal crown of spinulae as typical for the genus (Figs 7K, L, 8D, E). The whole antenna surface is covered by transverse rows of one to six flattened wide triangular spinulae (Figs 7K, L, 8D, E).

Antenna II typical for the genus (ANL/BL = 0.55–0.65), exopodite equal in length to endopodite or slightly longer (EXL/ENL = 1.0–1.1) (Fig. 7M–O). Armature of antenna II typical for the genus (Figs 7M–O, 8F, G).

Labrum and paragnaths typical for the genus (Fig. 7E, F, P).

Mandibles and first maxillae typical for the genus (Fig. 8H, I).

Five pairs of thoracic appendages, differing in size and structure, typical for the genus (Suppl. material 5: fig. S5). Gnathobase of limb II with eight to ten long filtering setae and four posterior elements (Fig. 7Q).

**Ephippial female.** Body length 0.8–1.0 mm. Body largely similar to that of parthenogenetic female (BL/BW = 1.8, BL/BH = 2.6–2.7), dorsal keel low (Fig. 9A, B). Ephippium ornamented by folds forming obscure irregular polygonal sculpture (Fig. 9A–C); well-visible polygons present only in ventral and posterior edge of the ephippium; between the large folds, ephippium surface covered by smaller wrinkles and pits (Fig. 9D). Morphology of head, thorax, abdomen, and postabdomen typical for the genus.

**Male.** Body length 0.61 mm, body oval in lateral view, moderately compressed laterally (BL/BH = 1.6, BL/BW = 2.3). Ventral margin slightly convex, armed by flattened setulae as typical for the genus (Fig. 10A–C). Posteroventral valve margin armed by groups of five to eight thin denticles alternating with solitary larger and thicker denticles (Fig. 10C, D); the dorsalmost portion of the posteroventral margin lacking well-defined groups of denticles (Fig. 10C). Head, thorax and abdomen morphology typical for the genus. Postabdomen shape and armature similar to that of parthenogenetic female (PL/BL = 0.2), postabdominal claw large and thick, slightly incurved, directed dorsally (PCL/PL = 0.4, PCL/PCW = 4.5) (Fig. 10E). Postabdominal setal length and armature typical for the genus. Two gonopores; each gonopore slit-like, located subdistally at the lateral surface of the postabdomen (Fig. 10E).

Antenna I is relatively short, almost straight, cylindrical in cross-section (AL/ED = 3.3, AL/AW = 6.9). Basal antennular seta similar to that of parthenogenetic female. Anterior antenna I face with additional male seta (ms) located proximally to the basal seta (Fig. 10F, H); the male seta is relatively short, 0.6× as long as the basal seta. Three clusters of four to six long thin setae at the anterior face of the appendage basally (Fig. 10F, H); three transverse rows of one to three flattened setae of various length and width more distally; two scale-like setae similar in size and location to that of parthenogenetic female (Fig. 10F, H). One additional scale-like seta at the posterior side of the antenna I, closely to its tip (Fig. 10G, H, black arrows). Distal armature of antenna I as in parthenogenetic female; appendage surface covered by transverse rows of two to three short wide triangular flattened spinulae.

Antenna II typical for the genus (ANL/BL = 0.64) (Fig. 10A). Distal basipodite end bearing two additional setae (mds) at outer face; one of setae 0.9–1.0× as long as the other, both setae naked (Fig. 10I).

Mouth parts typical for the genus.

Five pairs of thoracic appendages, differing in size and structure, typical for the genus.

Thoracic limb I largely similar to that of female (Fig. 8J–L, see Suppl. material 5: fig. S5A–F for female limb I morphology) but modified as typical for *L.rectirostris* male. IDL hook with a rounded tip almost reaching subdistal lobe (SDL); the hook tip bearing three subdistal transverse rows of very thin closely spaced spinulae at the anterior side (Fig. 8K). Subdistal lobe bearing five transverse rows of short closely spaced spinulae; the SDL surface lacking long setulae (Fig. 8L). Additional seta (X) located at anterior side of IDL (SX/S1 = 0.8). Additional anterior seta (Y) located at outer side of endite 3 (SY/S4 = 0.4).

Thoracic limbs II–V typical for the genus.

###### Differential diagnosis.

The observed differences between *Lathonurarectirostris* s. l. and *L.bekkerae* sp. nov. are summarized in Table 2. Both parthenogenetic and gamogenetic stages of *L.bekkerae* clearly differ from those of *L.rectirostris* s. l., first of all, by the armature of the posteroventral valve margin. Also, all studied specimens of *L.bekkerae* possess one or two additional posterior scale-like setae on the antennae I. Moreover, the specimens of *L.bekkerae* studied by scanning microscopy have a sculptured dorsal organ in contrast to *L.rectirostris* s. l. (Fig. 8C), although this character cannot be revealed when using optical microscopy. The ephippial female of *L.bekkerae* differs from that of *L.rectirostris* s. str. in the external ornamentation of the ephippium (irregular wrinkles forming obscure polygons versus well-distinct polygons in *L.rectirostris* s. str.). Finally, the male of *L.bekkerae* has a rather different armature of the limb I subdistal lobe, which bears long spinulae at the outer side (absent in males of *L.rectirostris* s. str.). However, male morphology was studied in one individual only, thus the latter features need to be confirmed.

**Table 2.** Diagnostic characters of *Lathonurarectirostris* (O.F. Müller, 1785) and *L.bekkerae* sp. nov.

Morphological character	*Lathonurarectirostris* (O.F. Müller, 1785)	*L.bekkerae* sp. nov.
Posteroventral valve margin	Uniform thin denticles	Groups of thin denticles alternating between solitary thick and long denticles
Antenna I, posterior scale-like setae	Absent	1–2
Dorsal organ	Smooth	Net-like sculpture
Ephippial female, ephippium ornamentation	Irregular polygons with almost smooth cells, clear polygonal sculpture	Wrinkles of different width and length, obscure polygonal sculpture
Male, groups of long spinulae at outer side of the SDL	Present	Absent

*Lathonurabekkerae* sp. nov. also differs from both species inquirendae of *Lathonura*, *L.dorsispina* and *L.ovalis*. *Lathonuradorsispina*, described from Romania, lacks figures in original description (Cosmovici 1901) and cannot be clearly attributed to *Lathonura* based on its diagnosis. The diagnostic features of *L.dorsispina* are: antenna II lacking transverse rows of denticles; numerous fine spinulae at “dorsal” (in fact probably referring to posterior) valve margin; small size (~0.4 mm, Cosmovici 1901). As noted above, valve morphology is unclear but, basing on description, seems to be more similar to *L.rectirostris* s. l. which has uniform closely spaced fine spinulae at posterior valve margin (Suppl. material 1: fig. S1A–F). Short spinulae present at antennae II of both *L.rectirostris* s. l. and *L.bekkerae* sp. nov. could easily have been missed by Cosmovici (1901). Finally, rather small individuals (~0.5 mm length) were also observed in both *L.rectirostris* s. l. and *L.bekkerae* sp. nov.

*Lathonuraovalis*, described from Pakistan (Mahoon and Sabjr 1985), can be attributed to the genus *Lathonura* basing on presence of flattened marginal setae at ventral valve margin and antenna II seta formula; however, most of characters included in the diagnosis of this taxon seem doubtful. The diagnostic features for this species are: incurved antenna I and straight postabdominal claw, seven “anal spines”, elliptical compound eye with only five ommatidia, six aesthetascs (Mahoon and Sabjr 1985: figs 43–47). None of these features can be referred to *L.bekkerae* and are observed in genus *Lathonura* in whole; it seems that Mahoon and Sabjr (1985) described a damaged or poorly fixed specimen. The individual depicted in Mahoon and Sabjr (1985: fig. 43) can be more likely attributed to *Macrothrix* based on body and antenna I shape and absence of flattened setae at ventral valve margin. Antenna II depicted in Mahoon and Sabjr (1985: fig. 45) is similar to that of *Lathonura* in seta formula, while some in Mahoon and Sabjr (1985: figs 44, 46, 47) cannot be referred to *Lathonura* with confidence.

###### Etymology.

The species is named after its collector, Evgenia I. Bekker, who strongly contributed to the studies of Eurasian cladocerans (Bekker et al. 2012, 2016, 2018).

###### Distribution and ecology.

*Lathonurabekkerae* sp. nov. is widely distributed in Northeast Asia, including Irkutsk Area, Zabaikalsky Krai, Yakutia, Magadan Area, and Kamchatka. Populations of *Lathonura* are relatively common in northeastern regions of Russia (Streletskaya 1975, 2010), and populations from southern Russian Far East (Kotov et al. 2011) and North China (Ji et al. 2015) likely belong to *Lathonurabekkerae* sp. nov. Note that *Lathonura* has not been observed in Korea (Jeong et al. 2014) and Japan (Uéno 1927), whereas records of “*L.rectirostris*” from South China apparently belong to *Guernellaraphaelis* Richard, 1892 (Ji et al. 2015). Also, *L.bekkerae* occurs in Alaska, but its distribution in North America remains unclear. Ecological preferences of the species have not been studied but seem to be rather similar to that of *L.rectirostris*. *Lathonurabekkerae* sp. nov. inhabits mostly vegetated lakes, ponds, and oxbows, but can be also found in vegetated zones of rivers, ditches, and creeks (Suppl. material 6).

## ﻿Discussion

### ﻿Morphological affinities and phylogenetic position of the genus *Lathonura*

Although *Lathonurarectirostris* was investigated previously by numerous authors, our study significantly updates and expands its description, especially as previous ones contained several inaccuracies and even contradictions concerning the thoracic limb morphology. For instance, several authors did not mention the presence of the accessory seta on thoracic limb I (Fischer 1848; Sergeev 1971, 1973; Fryer 1974; Silva-Briano 1998), but see Lilljeborg (1900) and Hudec (2010). This seta is often considered as a remnant of the thoracic limb I exopodite (Smirnov and Kotov 2010; Dumont 2016). Presence of one (very rarely two) accessory setae was observed in many anomopods, including the plesiomorphic families Gondwanotrichidae Van Damme, Shiel & Dumont, 2007, Dumontiidae Santos-Flores & Dodson, 2003 and Acantholeberidae Smirnov, 1976 (Smirnov 1976; Santos-Flores and Dodson 2003; Van Damme et al. 2007a, b; Smirnov and Kotov 2010; Dumont 2016). This feature of *Lathonura* seems to be unique (or, at least, rare) within the Macrothricidae (Silva-Briano 1998), although many representatives of the family require redescription.

Also, some authors depicted two normally developed ejector hooks of limb I (e.g., Silva-Briano 1998; Hudec 2010), while Fryer (1974) noted that they are completely absent in the studied specimens. In fact, one ejector hook of the limb I in *Lathonura* is small but normally developed, while the other one is reduced to a tubercle (Figs 5A, B, 8J, Suppl. material 5: fig. S5B, C). Most anomopod taxa have two normally developed ejector hooks (Kotov 1999; Kotov et al. 2004); complete absence of these structures was reported exclusively for *Neothrix* Gurney, 1927 (Dumont and Silva-Briano 1998), while in several representatives of *Macrothrix* Baird, 1843 and *Ilyocryptus* Sars, 1862 (Ilyocryptidae) only a single ejector hook is present (Kotov and Hollwedel 2004; Kotov et al. 2005; Garfias-Espejo et al. 2007; Korovchinsky et al. 2021), similar to *Lathonura*. As this morphological feature of *Lathonura* is not shared by all representatives of *Macrothrix* and *Ilyocryptus*, and these two genera are unlikely close relatives (Elmoor-Loureiro 2005; Kotov 2013; Van Damme et al. 2022), the loss of one or two ejector hooks seems to be an independent acquisition of several anomopod lineages, rather than synapomorphy of a particular group.

In addition to that of parthenogenetic females, we provide a detailed description of *Lathonura* gamogenetic stages. Males and females of *Lathonura* are clearly dimorphic, as known for other Macrothricidae (Smirnov 1976; Kořínek 1984; Kotov 1999; Silva-Briano and Dumont 2001; Dumont et al. 2002; Martinez-Caballero et al. 2017), despite the general male habitus being rather similar to that of the female. To date, a general comparison of male morphology among macrothricids is difficult as gamogenetic stages are poorly described for many taxa. *Lathonura* male antenna I bears additional setae on the anterior surface, similarly to a number of *Macrothrix* species having the anterior surface of the antennae I covered by rows of setulae (Dumont et al. 2002; Korovchinsky et al. 2021). Antenna II basipodite bears two additional setae, which were observed in *Drepanothrix* Sars, 1862 (Dadykin, unpublished), *Grimaldina* Richard, 1892 (Kořínek 1984: pl. XXVI, fig. 6) and *Guernella* Richard, 1892 (Kořínek 1984: pl. XXVII, fig. 5), but not in other Macrothricidae (Kotov 2013). Thoracic limb I modification pattern in *Lathonura* male is rather similar to *Pseudomoina* Sars, 1912 (Smirnov 1976), *Drepanothrix* (Dadykin, unpublished), *Macrothrixtripectinata* Weisig, 1934 (Kotov 1999), and *Guernella* (Kořínek 1984) in having subdistal lobe at anterior limb surface. This lobe, however, bears none to two setae in different genera, indicating possible adaptive origin of these structures. Based on its position, the subdistal lobe is likely to participate in female capturing during copulation. No subdistal lobe was observed in males of other Macrothricidae (Smirnov 1976; Kořínek 1984; Silva-Briano and Dumont 2001; Dumont et al. 2002; Martinez-Caballero et al. 2017). Finally, the postabdomen of *Lathonura* male is weakly modified in comparison to that of the female, which is typical for macrothricids (Smirnov 1976; Kotov 1999; 2008; Silva-Briano and Dumont 2001).

To sum up, male and parthenogenetic female morphology of *Lathonura* displays several traits unique or rare among Macrothricidae, as well as some common features. The position of *Lathonura* within the family has already been argued by several authors (Dumont and Silva-Briano 1998; Silva-Briano 1998; Elmoor-Loureiro 2005). Unlike the ‘core’ group of macrothricids (*Macrothrix*, *Wlassicsia* Daday, 1904, *Bunops* Birge, 1893, *Streblocerus* Sars, 1862), *Lathonura* and some other genera have simple (not forked) anterior setae on limb I (Dumont and Silva-Briano 1998). Based on the structure of thoracic limb IV and presence of a swimming seta on the basal segment of antenna II exopodite, *Lathonura* was supposed to be related to *Neothrix*, *Guernella* and *Pseudomoina* (Elmoor-Loureiro 2005). It should be noted that some of the features used for the latter cladistic analysis were mistakenly attributed to *Lathonura* (Elmoor-Loureiro 2005), e.g., presence of two ejector hooks or presence of six or seven filtering setae of gnathobase II (up to 10 in *L.bekkerae* sp. nov.). This is also true for another attempt to reconstruct the phylogeny of Macrothricidae based on morphological features (Dumont and Silva-Briano 1998). Therefore, the phylogenetic position of *Lathonura* should be clarified by further molecular and comparative morphological analysis.

Our morphological analysis supports a subdivision of the Holarctic *Lathonura* into at least two morphospecies, *L.rectirostris* s. l. and *L.bekkerae* sp. nov. The new species is morphologically distinct from both *L.dorsispina* and *L.ovalis*, although their descriptions are very incomplete (see the Differential diagnosis section in Results). The differences in posteroventral valve armature observed between *L.rectirostris* and *L.bekkerae* sp. nov. might affect male attachment during copulation and be efficient for preventing hybridization, as supposed for *Chydorus* Leach, 1816 (Van Damme and Dumont 2006). Similar differences were observed in a number of Anomopoda and Ctenopoda Sars, 1865, including *Moina* Baird, 1850 and *Diaphanosoma* Fischer, 1850 (Korovchinsky 2018; Korovchinsky et al. 2021). However, to date copulatory behavior has been studied for a few anomopod species only (Brewer 1998; Winsor and Innes 2002; Van Damme and Dumont 2006). Also, the two species differ by gamogenetic stage morphology, a pattern rarely observed among macrothricids and known to date only for *Bunops* and several *Macrothrix* species, although insufficiently studied (Smirnov 1976; Kotov 1999, 2008; Silva-Briano and Dumont 2001; Dumont et al. 2002; Martinez-Caballero et al. 2017). Nevertheless, differentiation of close taxa within a species complex by gamogenetic stage morphology is rather common for Anomopoda in general (Korovchinsky et al. 2021).

### ﻿Morphological and genetic diversity of the genus *Lathonura*

Barcoding of mitochondrial gene fragments (Hebert et al. 2003) has proven to be a powerful method for investigations of Cladocera genetic structure and species revisions (Belyaeva and Taylor 2009; Bekker et al. 2012; Zuykova et al. 2018). In the case of *Lathonura*, analysis of the COI Folmer fragment conducted in this study confirms a separate status of *L.bekkerae* sp. nov. It should be noted that populations of L.cf.rectirostris s. l. from North Canada and, probably, the Atlantic Coast of U.S.A. belong to a particular, deeply divergent lineage within clade A1 (Fig. 2) and might represent a separate taxon. Although no morphological differences were observed between parthenogenetic individuals of European *L.rectirostris* s. str. and East American *L.rectirostris* s. l., they might differ in morphology of gamogenetic stages which were not studied for the American populations. In this respect, distribution, and genetic diversity of *Lathonura* in the Nearctic requires further revisions.

*Lathonurarectirostris* s. l. and *L.bekkerae* sp. nov. display a characteristic ‘non-cosmopolitan’ geographic pattern (Frey 1982; Forró et al. 2008; Kotov 2016) previously observed in many genera or species-groups of Branchiopoda (Xu et al. 2009, 2011; Kotov et al. 2016; Neretina and Kotov 2017b; Bekker et al. 2018; Taylor et al. 2020; Garibian et al. 2021). The known range of *L.bekkerae* includes Northeast Eurasia (east to Taimyr Peninsula) and Alaska, which fits well with the existing model of Cladocera biogeography (Korovchinsky 2004; Kotov 2016; Kotov et al. 2016). For several branchiopod groups, Central Siberia (line ‘Baikal-Taimyr’) is a boundary separating ranges of closely related species (Belyaeva and Taylor 2009; Millette et al. 2011; Kotov et al. 2016; Bekker et al. 2016, 2018; Sinev and Gavrilko 2021; Zuykova et al. 2022), although the possible reason for this separation is still discussed. Most probably, the faunistic differentiation between West and East Eurasian anomopod fauna is a consequence of Pleistocene glaciation, as ice shield formed mostly on the western part of the continent (Hewitt 2004; Brubaker et al. 2005; Anderson et al. 2010). In a number of cladoceran species, East Asian populations demonstrate a significantly higher genetic divergence in comparison to those from West Eurasia, which could be explained by rapid post-Pleistocene colonization of these, previously glaciated, areas from refugia in East Asia (Millette et al. 2011; Zuykova et al. 2018, 2019, 2022). Anyway, available genetic data are insufficient for reconstructing a detailed phylogeography of *Lathonura* in the northern temperate zone.

Moreover, although the main range of *Lathonura* lies in the North Holarctic, there are a few records of the genus away from this zone. The records of *Lathonura* in Azerbaijan (Behning 1941), West Uzbekistan (Akatova 1950) and Pakistan (Mahoon and Sabjr 1985) might refer to *L.rectirostris* s. str. and indicate a southern distribution border for this taxon. Also, representatives of the genus were observed in subtropical regions of Africa – in Botswana (Hart and Dumont 2005) and the Republic of South Africa (Smirnov 2008; Kotov, unpublished). Preliminary analysis shows that the parthenogenetic females from South Africa share the posteroventral valve margin morphology with European *L.rectirostris* s. str. In this respect, to date the origin and status of the African populations is unclear. First, they might be recent invaders from the Palearctic, which was proved for many Cladocera (Kotov et al. 2022). On the contrary, ephippia of *Lathonura*, being normally attached to substrate (Fryer 1974), are unlikely to spread across large distances, thus the African populations could belong to a particular species inhabiting the tropics and subtropics of Africa, as was shown for a number of anomopods (Van Damme and Eggermont 2011; Van Damme et al. 2013; Neretina and Kotov 2015). Unfortunately, our knowledge about African Anomopoda is still rather scarce (Forró et al. 2008; Smirnov 2008; Van Damme et al. 2013; Etilé et al. 2020). Thereby, the distribution of *Lathonura* in tropical and subtropical regions of the Old World still requires further studies, as well as taxonomy of the genus and its position compared to other macrothricids in general.

## ﻿Conclusions

Despite former morphological, systematic and faunistic research, many aspects of *Lathonura* taxonomy, phylogeny, and ecology still remain unexplored. Here, we show that even in Eurasia morphological and genetic diversity of *Lathonura* is higher than previously known. We hope that our study will promote future research of these remarkable animals and their relatives.

## Supplementary Material

XML Treatment for Lathonura

XML Treatment for Lathonura rectirostris

XML Treatment for Lathonura bekkerae

## ﻿Additional information

### Use of AI

No use of AI was reported.

### Author ORCIDs

Ivan A. Dadykin https://orcid.org/0000-0002-7356-5884

Dmitry D. Pereboev https://orcid.org/0009-0004-8194-7585

### Data availability

All of the data that support the findings of this study are available in the main text or Supplementary Information. Original DNA sequences are available in the NCBI GenBank database (access numbers PV430703–PV430727).

## Supplementary materials

Supplementary material 1 Supplementary figure S1 Data type tif Explanation note Postero-ventral valve armature in different populations of *Lathonura* Lilljeborg, 1853. A–F. *Lathonurarectirostris* (O.F. Müller, 1785) s. l.: A. Innlandet, Norway (loc. 1); B. Nizhny Novgorod Area, Russia (loc. 11); C. Chelyabinsk Area, Russia (loc. 15); D. Tomsk Area, Russia (loc. 17); E. Uvs Province, Mongolia (loc. 18); F. New Hampshire, USA (loc. 20); G–J. *Lathonurabekkerae* sp. nov.: G. Alaska, USA (loc. 34); H. Yakutia, Russia (loc. 27); I. Kamchatsky Krai, Russia (type locality, loc. 31); J. Zabaikalsky Krai, Russia (loc. 26). Scale bars: 20 µm. For information on localities, see Fig. 1, Table 1. https://binary.pensoft.net/file/1397535This dataset is made available under the Open Database License (http://opendatacommons.org/licenses/odbl/1.0/). The Open Database License (ODbL) is a license agreement intended to allow users to freely share, modify, and use this Dataset while maintaining this same freedom for others, provided that the original source and author(s) are credited.Ivan A. Dadykin, Dmitry D. Pereboev

Supplementary material 2 Supplementary figure S2 Data type tif Explanation note *Lathonurarectirostris* (O.F. Müller, 1785) s. str., parthenogenetic female from Lake Randsfjorden, Innlandet, Norway (Fig. 1, loc. 1), scanning electron microscopy. A. General lateral view; B, C. Dorsal organ; D. Frontal head pore, anterior view; E–H. Antenna I: E. Outer view (white arrow shows absence of posterior scale-like setae); F. Anterior view; G. Anterior scale-like setae; H. Aesthetasc tip; I–M. Antenna II: I. General outer view; J. Basal sensory setae; K. Exopodite, inner view; L. Exopodite segment 1 swimming seta; M. Endopodite, inner view. Abbreviations: aes, aesthetascs; bss, basal sensory setae; dbs, distal burrowing spine; do, dorsal organ; en, endopodite; ex, exopodite; scs, scale-like setae. White arrows indicate diagnostic features. Scale bars: 200 µm (A); 50 µm (B, C, M); 10 µm (D, H); 20 µm (E, F, I, J, K); 10 µm (G, L). https://binary.pensoft.net/file/1397536This dataset is made available under the Open Database License (http://opendatacommons.org/licenses/odbl/1.0/). The Open Database License (ODbL) is a license agreement intended to allow users to freely share, modify, and use this Dataset while maintaining this same freedom for others, provided that the original source and author(s) are credited.Ivan A. Dadykin, Dmitry D. Pereboev

Supplementary material 3 Supplementary figure S3 Data type tif Explanation note *Lathonurarectirostris* (O.F. Müller, 1785) s. str. from Lake Randsfjorden, Innlandet, Norway (Fig. 1, loc. 1), scanning electron microscopy. A–E. Parthenogenetic female: A. Right mandible; B. Left mandible; C. Posteroventral valve margin (white arrows show uniform denticles); D. Marginal seta; E. Valve marginal denticles; F–H. Ephippial female: F. Lateral view; G. Dorsal view; H. Ephippium sculpture. Abbreviations: a, anterior mandible side; p, posterior mandible side; pdt, posterodorsal mandible teeth. Scale bars: 10 µm (A, B, D); 50 µm (C, H); 2 µm (E); 200 µm (F, G). https://binary.pensoft.net/file/1397537This dataset is made available under the Open Database License (http://opendatacommons.org/licenses/odbl/1.0/). The Open Database License (ODbL) is a license agreement intended to allow users to freely share, modify, and use this Dataset while maintaining this same freedom for others, provided that the original source and author(s) are credited.Ivan A. Dadykin, Dmitry D. Pereboev

Supplementary material 4 Supplementary figure S4 Data type tif Explanation note *Lathonurarectirostris* (O.F. Müller, 1785) s. str., male from Lake Randsfjorden, Innlandet, Norway (Fig. 1, loc. 1), scanning electron microscopy. A, B. Postabdomen: A. General lateral view; B. Distal postabdomen portion (postabdominal claws are broken out); C–E. Antenna I (white arrows indicate additional anterior setae): C. Outer view; D. Anterior view; E. Antenna I base, anterior view; F, G. Antenna II, distal basipodite setae; H–M. Thoracic limb I: H. General outer view; I. Subdistal lobe and IDL hook in outer view; J. Distal IDL portion in outer view; K. Armature of the IDL seta 1; L, M. Subdistal lobe armature. Abbreviations: acs, accessory seta of the limb I; ao, anal opening; bp, basipodite; bss, basal sensory seta of antenna I; dbs, distal burrowing spine; en, endopodite; ex, exopodite; gp, gonopore; h, IDL hook; IDL, inner distal lobe of the limb I; mds, male distal basipodite setae; ODL, outer distal lobe of the limb I; PI, PII, thoracic limbs I and II; pam, preanal postabdomen margin; ppr, postabdominal process; scs, scale-like setae; SDL, subdistal lobe of the limb I. Scale bars: 50 µm (A, C, D, F); 20 µm (B, E, G–J, L); 5 µm (K, M). https://binary.pensoft.net/file/1397538This dataset is made available under the Open Database License (http://opendatacommons.org/licenses/odbl/1.0/). The Open Database License (ODbL) is a license agreement intended to allow users to freely share, modify, and use this Dataset while maintaining this same freedom for others, provided that the original source and author(s) are credited.Ivan A. Dadykin, Dmitry D. Pereboev

Supplementary material 5 Supplementary figure S5 Data type tif Explanation note *Lathonurabekkerae* sp. nov., thoracopods of parthenogenetic female from the type locality (Fig. 1, loc. 31), scanning electron microscopy. A. Intact thoracic limbs I–V, ventral view; B–F. Thoracic limb I: B. Outer view; C. Ejector hooks in outer view (white arrow shows a reduced ejector hook); D. IDL in anterior view; E. Endites 2 and 3, anterior surface; F. Anterior setae 3’ and 4’; G, H. Thoracic limb II: G. Exopodite in posterior view; H. Scraping setae; I. Gnathobase IV. Abbreviations: e1–3, endites 1–3; ejh, ejector hooks; gnII–IV, gnathobase II–IV; IDL, inner distal lobe of the limb I; ODL, outer distal lobe of the limb I; PI–PV, thoracic limbs I–V. Scale bars: 100 µm (A, B); 10 µm (C); 50 µm (D, E); 20 µm (F–I). https://binary.pensoft.net/file/1397539This dataset is made available under the Open Database License (http://opendatacommons.org/licenses/odbl/1.0/). The Open Database License (ODbL) is a license agreement intended to allow users to freely share, modify, and use this Dataset while maintaining this same freedom for others, provided that the original source and author(s) are credited.Ivan A. Dadykin, Dmitry D. Pereboev

Supplementary material 6 List of studied localities and specimens Data type xlsx Explanation note Locality numbers correspond to the numbers in Fig. 1. Asterisk indicates a record obtained from GenBank that was not included in morphological analysis. https://binary.pensoft.net/file/1397540This dataset is made available under the Open Database License (http://opendatacommons.org/licenses/odbl/1.0/). The Open Database License (ODbL) is a license agreement intended to allow users to freely share, modify, and use this Dataset while maintaining this same freedom for others, provided that the original source and author(s) are credited.Ivan A. Dadykin, Dmitry D. Pereboev
